# Immunomodulation Strategies for the Successful Regeneration of a Tissue‐Engineered Vascular Graft

**DOI:** 10.1002/adhm.202200045

**Published:** 2022-03-27

**Authors:** Fan Zhang, Martin W. King

**Affiliations:** ^1^ Wilson College of Textiles North Carolina State University Raleigh NC 27606 USA

**Keywords:** immune response, immunomodulation, macrophage, tissue engineering, tissue‐engineered vascular grafts

## Abstract

Cardiovascular disease leads to the highest morbidity worldwide. There is an urgent need to solve the lack of a viable arterial graft for patients requiring coronary artery bypass surgery. The current gold standard is to use the patient's own blood vessel, such as a saphenous vein graft. However, some patients do not have appropriate vessels to use because of systemic disease or secondary surgery. On the other hand, there is no commercially available synthetic vascular graft available on the market for small diameter (<6 mm) blood vessels like coronary, carotid, and peripheral popliteal arteries. Tissue‐engineered vascular grafts (TEVGs) are studied in recent decades as a promising alternative to synthetic arterial prostheses. Yet only a few studies have proceeded to a clinical trial. Recent studies have uncovered that the host immune response can be directed toward increasing the success of a TEVG by shedding light on ways to modulate the macrophage response and improve the tissue regeneration outcome. In this review, the basic concepts of vascular tissue engineering and immunoengineering are considered. The state‐of‐art of TEVGs is summarized and the role of macrophages in TEVG regeneration is analyzed. Current immunomodulatory strategies based on biomaterials are also discussed.

## Graphical abstract

Recent studies start to shed light on ways to modulate the macrophage response toward increasing the success of a tissue‐engineered vascular graft (TEVG). This review introduces the basic concepts of vascular tissue engineering and immunoengineering and highlights current immunomodulatory strategies based on biomaterials. The state‐of‐art of TEVGs is summarized, and the role of macrophages in TEVG regeneration is also analyzed.

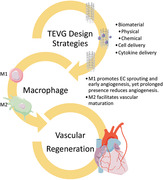

## 1 Introduction

Cardiovascular disease is the leading cause of death all over the world. Despite urgent needs of viable vascular grafts, there is no clinically relevant graft approved and available on the market yet. A variety of avenues to build a viable tissue‐engineered vascular graft (TEVG) have been attempted, but we are still far away from bringing up a reliable alternative to the autologous graft. Researchers have explored various biomaterials, cells and cytokines to improve TEVG remodeling, which has laid a solid background for the development of TEVGs.

However, in only recent years, have researchers started to shed light on the role of immune cells, which are the first responders to the TEVG after implantation. In 2010, Roh et al. unveiled the finding that a TEVG remodels in an inflammation‐mediated way by the recruitment of monocytes.^[^ ^1^ ^]^ Following that, increasing evidence implicated the important role of inflammation in graft remodeling. We then started to realize the immune cells are those which set up a microenvironment for subsequent cell infiltration, migration, proliferation, and differentiation. It remains obscure about the specific mechanism of immune cells regulating graft remodeling and therefore more studies are needed to explore this phenomenon.

In this review, we have reviewed recent advances at the interface of immunoengineering and vascular tissue engineering. We have further evaluated the state of art of TEVG development and recent advances in understanding the role of inflammation in TEVG remodeling. We then summarize biomaterial‐based immunomodulatory strategies in promoting vascular tissue regeneration and analyzed how they can be integrated and applied in building a successful TEVG.

### 1.1 Coronary Artery Disease and Coronary Artery Bypass Graft

Coronary artery disease (CAD) is a key component contributing to cardiovascular disease, which is the leading cause of death worldwide.^[^ ^1^, ^2^ ^]^ CAD is characterized by narrowing, rupture, or blockage of the coronary artery due to atherosclerosis in the blood vessel, resulting in inadequate blood supply to heart muscle. The most common symptoms include angina pectoris, myocardial infarction, and ischemic heart failure,^[^ ^2^ ^]^ all of which are painful and lethal.

The risk factors for CAD include genetic defects and environmental risks, behavioral factors such as an unhealthy diet, low level of physical activity, smoking, and alcohol abuse, as well as high blood pressure, high blood glucose and lipid, and other chronic conditions such as diabetes, obesity, autoimmune diseases, and aging.^[^ ^3^ ^]^

Depending on the severity of the disease, the treatments of CAD range from lifestyle changes, medications, angioplasty to surgery. The most commonly used revascularization surgery for CAD is coronary artery bypass graft (CABG) surgery and percutaneous coronary intervention (PCI). CABG has a longer history and is more effective in reducing the risk of cardiac infarction by restoring adequate blood flow to ischemic heart muscle in stable CAD.^[^ ^2^ ^]^ However, CABG requires open‐heart surgery. It is recommended for patients with multivessel disease, left main CAD or with type 1 diabetes due to its superior short‐term and long‐term (>5 years) outcome^[^ ^2^, ^4^, ^5^ ^]^ PCI is a less invasive alternative for less complex CADs.^[^ ^6^ ^]^ It does not require open heart surgery. However, the use of bare metal stents to open occluded vessels has shown a 15–60% in‐stent restenosis in 1 to 2 years after implantation.^[^ ^7^ ^]^ Even with the newer‐generation of drug‐eluting stents that reduced the risk of acute thrombosis, incomplete revascularization, and late‐stage restenosis remain problematic.^[^ ^8^, ^9^ ^]^ Studies are ongoing to improve the stent, such as reducing the strut thickness and adding a biodegradable drug‐releasing coating. It is anticipated that these improvements will reduce the risk of restenosis of the stents.^[^ ^10^, ^11^ ^]^

**Figure 1:**
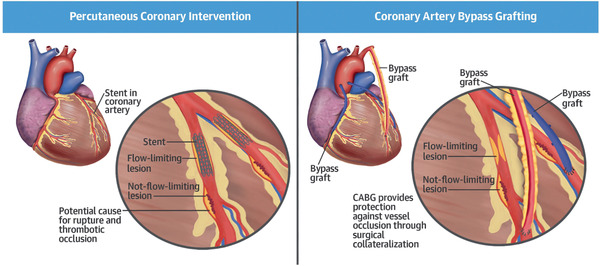
Surgery treatment methods of coronary artery disease includes coronary artery bypass surgery and percutaneous coronary intervention. Reproduced with permission.^[^ ^2^ ^]^ Copyright 2022, Elsevier.

An advantage of CABG over PCI was pointed out by Doenst et al. that PCI is only able to ameliorate flow‐limiting stenosis.^[^ ^2^ ^]^ However, there are also nonflow‐limiting stenosis that are at risk of rupture and cause thrombotic occlusion and further cardiac infarction as shown in **Figure** **1**. Only CABG is able to protect patients from both types of stenoses since they are delivered distal to the plaque and provide a collateral flow.^[^ ^2^ ^]^

There are vast numbers of patients requiring CABG surgery each year and therefore significant needs for CABGs. The current gold standard for CABG is to use an autologous graft, which is the artery or vein from the patients’ own body.^[^ ^12^ ^]^ The saphenous vein (SV) is the most transplanted graft in CABG surgery. However, this requires a secondary surgical site in order to harvest the graft.^[^ ^13^ ^]^ In addition, there are more than 30% of patients who do not have an SV available due to their systemic vascular disease or to previous harvesting.^[^ ^7^ ^]^ Vein grafts fail in 40–50% of the patients by 10 years after implantation.^[^ ^14^ ^]^ The main reasons are the stenosis of the graft caused by intimal hyperplasia, graft remodeling, and thrombosis.^[^ ^7^, ^12^, ^14^ ^]^ Internal mammary artery is another option for an autologous graft with 10‐year patency rate greater than 90%,^[^ ^12^ ^]^ but sometimes it is not long enough.^[^ ^7^ ^]^ Although autologous grafts are the current gold standard, there are around 20% patients who do not have an available graft.^[^ ^15^ ^]^ In addition, according to clinical data, around 15% of the autologous bypass grafts result in narrowing (stenosis) or occlusion within 1 year after implantation.^[^ ^13^ ^]^

Synthetic arterial prostheses made from polyethylene terephthalate or expanded polytetrafluoroethylene (ePTFE) present high failure rates when the diameter is smaller than 6 mm due to intimal hyperplasia, thrombosis, low patency, and mechanical compliance mismatch.^[^ ^7^ ^]^ On the other hand, revascularization is also involved in the treatment of pediatric patients with congenital cardiovascular defects (CCDs).^[^ ^16^ ^]^ Pediatric CCDs are reported in 1% of live births.^[^ ^13^ ^]^ Synthetic vascular prostheses cause a high rate of morbidity and mortality.^[^ ^13^ ^]^ For pediatric patients, synthetic grafts lack growth potential, and require secondary or multiple interventions during their lifetime.^[^ ^17^ ^]^ Not only coronary artery bypass surgery, but also peripheral popliteal and carotid bypass surgery lacks viable small diameter (<6 mm) vascular grafts.

Given the severe shortage of small diameter arterial grafts and the limitations of autologous and synthetic grafts, there is a significant need for alternative solutions. The TEVG has arisen as a promising solution with better compatibility and growth potential. Not only used in CABG, small‐diameter TEVGs are also in great need for peripheral vascular disease, carotid artery bypass, and renal dialysis purposes.^[^ ^15^ ^]^ A vast amount of work has been dedicated for the development of TEVGs, but the progress is slow. TEVGs are typically fabricated by biodegradable synthetic or natural polymers so that the scaffold materials degrade at the same time as neotissue ingrowth, which gradually transforms the mechanical load to the new tissue.^[^ ^17^ ^]^ Ultimately, the scaffold will be replaced by the newly formed tissue to form a native‐like vessel that has similar physiological properties and growth potential.

However, in order to advance the TEVG to clinical practice, there is still a long way to go because of graft thrombosis and stenosis postoperatively.^[^ ^17^ ^]^ Researchers have spent years to optimize the scaffold design and cell encapsulation. However, another critical controllable component has been overlooked—the immune system, the first responder to the TEVG postimplantation.

Increasing amounts of evidence uncover the critical role of immune cells in the success of TEVGs, and more and more studies start to shed light on modulating the immune response for tissue regeneration purposes. Immune cells secrete cytokines that set up a microenvironment for the subsequent tissue regeneration or even directly participate in the regeneration process.^[^ ^18^ ^]^ Instead of avoiding the detection by the immune system, recent research has started to appreciate the role of the immune system in the remodeling process and tried to direct immune cells to set up a pro‐regeneration microenvironment to promote tissue remodeling in a preferred direction.

### 1.2 Interface of Immunomodulation and Tissue Engineering

#### 1.2.1 Brief Overview of the Role of Immunology in Tissue Engineering

The timing of cell recruitment affects the tissue regenerative process.^[^ ^19^ ^]^ Immune cells arrive at the biomaterials in the very early‐stage post implantation and set up a microenvironment for the subsequent wound healing and tissue regeneration. And thus, it is important to modulate the immune cells so as to set up a pro‐regenerative environment for the preferred tissue engineering process to happen. Especially in the development of atherosclerosis, the immune system is extensively involved; however, the role of immune response in engineering vascular tissue has not yet been extensively explored.

**Figure 2:**
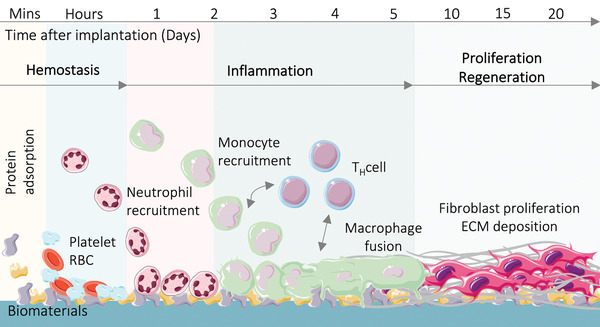
Timeline of early cell recruitment to biomaterials after implantation. Immediately after biomaterial implantation, proteins are adsorbed on the material surface followed by the adhesion of platelets and blood cells. The innate immune system response causes a rapid influx of neutrophils and macrophages, which interact with each other, T helper cells and other signals in the microenvironment. The foreign body response occurs later when macrophages fuse into foreign body giant cells and fibroblasts produce fibrous tissue to encapsulate foreign materials. The *x*‐axis represents time and the *y*‐axis represents the relative distance of cells away from the biomaterial. Produced using the cell templates from Servier Medical Art under the Creative Commons Attribution 3.0 Unported License.

As shown in **Figure** **2**, immediately after implantation, a variety of proteins in the interstitial fluid or blood are adsorbed onto the biomaterials surface, forming a corona or a provisional matrix around the scaffold,^[^ ^20^ ^]^ which will directly influence the body's response to the material.^[^ ^21^ ^]^ Platelets and red blood cells also arrive in a short time to prevent hemorrhage and form a temporary scaffold for cell recruitment.^[^ ^20^, ^22^ ^]^

The presence of damaged cells will lead to tissue released damage‐associated molecular patterns (DAMPs). If contamination, such as bacteria, parasites, and viruses, is present at the wound site, there will be pathogen‐associated molecular patterns (PAMPs). DAMPs and PAMPs can be recognized by the stroma cells and tissue‐resident immune cells, which will release relevant cytokines and chemokines to elicit acute inflammation by the innate immune system.^[^ ^23^ ^]^

Neutrophils are the first responder among the innate immune cells. They are recruited to the wound site to break down dying cells and contamination, clear the cellular debris by phagocytosis and degrade the extracellular matrix (ECM) with a protease enzyme. Neutrophils release cytokines, such as interferon‐*γ* (IFN‐*γ*) and interleukin‐10 (IL‐10), which mediate macrophage and T helper cell (T_H_) activity.^[^ ^23^ ^]^ They also participate in angiogenesis by releasing vascular endothelial growth factor (VEGF), transforming growth factor‐*β* (TGF‐*β*) and regulating tissue remodeling by secreting matrix metalloproteinases (MMPs). However, neutrophils are only present during the early stage of inflammation. Their prolonged presence will lead to a continuous tissue response and chronic inflammation.^[^ ^23^ ^]^

Macrophages arrive at the wound site immediately following the neutrophils,^[^ ^24^ ^]^ and they work synergistically to clear any cellular debris and contamination. Macrophages either reside in the tissue or are differentiated from monocytes in the circulation.^[^ ^23^ ^]^ Circulating monocytes sense recruitment signals through monocyte chemoattractant proteins (MCPs) receptors and C–C chemokine receptor 2 (CCR2) molecules,^[^ ^23^ ^]^ and rapidly differentiate into macrophages after they infiltrate into the adjacent tissue.^[^ ^24^ ^]^ During the different stages of inflammation, macrophages show different phenotypes: the early proinflammatory type 1 (M1) phenotype, and later the anti‐inflammatory, prohealing or pro‐regenerative type 2 (M2) phenotype. The role of the macrophage is actively studied at the interphase between immunoengineering and tissue engineering, which will be discussed further in Section 2.2. Other innate cells, such as neutral killer (NK) cells and dendritic cells, are not extensively involved in the biomaterial mediated tissue regenerative process.^[^ ^23^ ^]^

The early recruitment of these innate immune cells at the biomaterial surface is critical to the ultimate level of tissue regeneration. The low level of early cell recruitment retards subsequent tissue cell recruitment and proliferation. Yet excessive cell recruitment can lead to early narrowing or stenosis of the vascular graft and rapid scaffold remodeling, which will adversely affect the long‐term graft integrity and mechanical properties.^[^ ^25^, ^26^, ^27^ ^]^ The recruited cells respond to different biomaterial substrates and set up a microenvironment where cell proliferation and differentiation involves the release of a series of cytokines and chemokines.

Adaptive immune cells do not necessarily respond to biomaterial‐mediated tissue regeneration. But CD4^+^ T helper cells (T_H_) and regulatory T cells (T_reg_) have been found to crosstalk innate immune cells into regulating tissue repair. T_H_1 cells crosstalk with macrophage to induce the M1 phenotype whereas T_H_2 cells induce the M2 phenotype. T_H_17‐related factors result in neutrophil recruitment and proliferation.^[^ ^23^ ^]^ T_reg_ cells and NK cells have also been found to mediate postnatal neovascularization.^[^ ^1^ ^]^ For example, any T_reg_ cells present suppress antigen‐specific T cell function, induce neutrophil apoptosis, release anti‐inflammatory molecules, and guide macrophages toward the M2 phenotype. T_reg_ cells also modulate stromal or progenitor cells in the regeneration process.^[^ ^23^ ^]^

Gamma delta T cells (*γδ* T cells), which are innate‐immune‐cell‐like T cells that express the *γδ* T cell receptor, also participate in tissue regeneration by releasing the insulin‐like growth factor (IGF)‐1 and interleukin (IL)‐17 that promotes macrophage recruitment.^[^ ^23^ ^]^ It has also been found that tissue‐resident *γδ*T cells enhance cell function and injury repair.^[^ ^28^ ^]^

As illustrated in Figure 2, without a successful rapid resolution of the acute inflammatory reaction, macrophages can fuse and form multinucleated foreign body giant cells (FBGCs) as a part of the foreign body response. This process can promote fibrotic tissue formation and even failure of an implant.^[^ ^29^, ^30^ ^]^ It happens particularly when a synthetic material is implanted. FBGCs form to degrade the implanted material by releasing enzymes and reactive oxygen species.^[^ ^30^ ^]^ Around 7 days after implantation, fibroblasts are recruited to the site and deposit fibrous collagen to encapsulate the implanted biomaterial, which will then adversely affect the compliance of the material and hinder the tissue regeneration process. The formation of FBGCs and fibrotic capsules around a biomaterial implant can be mediated by many factors, including, but not limited to, material morphology,^[^ ^31^ ^]^ composition,^[^ ^32^ ^]^ and stiffness.^[^ ^33^ ^]^

#### 1.2.2 Monocytes and Macrophage

As we mentioned above, host monocytes and macrophages are among the first responders to a biomaterial implant and are extensively involved in the TEVG remodeling process. Excessive infiltration of macrophages into the scaffold leads to stenosis of the graft but insufficient infiltration retards neotissue formation and the graft regeneration process.^[^ ^25^, ^26^ ^]^

**Figure 3:**
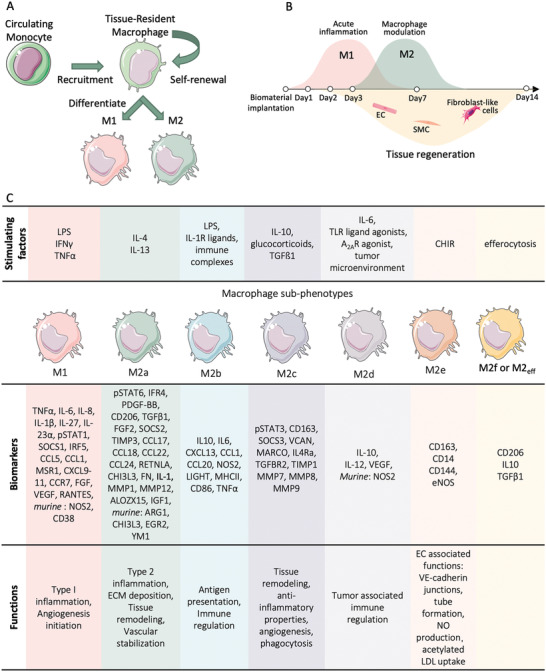
Macrophage differentiation. A) Macrophages can be recruited from circulating monocytes or from the surrounding tissue. B) Macrophages are recruited to the biomaterials earlier than tissue‐forming cells and play a crucial role in setting up the immune microenvironment that mediates the tissue regeneration process. C) Different phenotypes of macrophages are developed in response to various stimulating factors. They stain positive to distinct biomarkers and assume different functions. Produced using the cell templates from Servier Medical Art the Creative Commons Attribution 3.0 Unported License.

Macrophages are heterogenous phagocytes that perform various functions according to their location.^[^ ^34^ ^]^ This might result from their exposure to a distinct array of molecular signals in different tissues (**Figure** **3**).^[^ ^34^ ^]^ Macrophages have different phenotypes that since the year 2000 have been classified into the M1 and M2 phenotype.^[^ ^34^, ^35^ ^]^

The M1 phenotype is generally considered to be classically activated and proinflammatory.^[^ ^35^ ^]^ It primarily participates in phagocytosis and angiogenesis.^[^ ^23^ ^]^ However, the prolonged presence of the M1 macrophage leads to chronic inflammation that last for weeks, which will hinder tissue repair. The M1 phenotype can be induced by exposure to IFN‐*γ*, lipopolysaccharide (LPS), and tumor necrosis factor alpha (TNF‐*α*), which can lead to the production of IL‐12, nitric oxide (NO), and IL‐23.^[^ ^36^ ^]^

In addition, M1 macrophages release VEGF at the early stage after biomaterial implantation, establishing an immature neovascular network.^[^ ^37^ ^]^ Dondossola et al. found that this early initiation and maintenance of the neovasculature was associated with the formation of dense fibrotic encapsulation around their electrospun polycaprolactone (PCL) scaffold.^[^ ^37^ ^]^ This fibrotic capsule around the implant can hinder mass transport and electrical communication^[^ ^38^ ^]^ and can lead to functional impairment and even organ failure.^[^ ^35^ ^]^ TNF‐*α* released by M1 macrophages has also been observed to induce osteoblastic differentiation and mineralization of the cells isolated from the vascular medial layer.^[^ ^39^ ^]^

On the other hand, M2 macrophages are generally recognized as activated alternatively through an anti‐inflammatory and pro‐regenerative pathway. They can stabilize angiogenesis and promote ECM remodeling.^[^ ^23^ ^]^ M2 macrophages can be further divided into subtypes, including M2a, M2b, M2c, M2d, M2e, each of them having a distinct function as shown in Figure 3.^[^ ^18^, ^36^ ^]^

Cha et al. found that the activation of the M2 phenotype depends on an integrin‐mediated pathway.^[^ ^40^ ^]^ Thus, biomaterials with the appropriate cell binding motifs would prefer to resolve any inflammation and promote tissue regeneration. They clearly showed in an in vitro study that a gelatin methacryloyl (GelMA) hydrogel with cell binding sites polarized THP‐1 cells more easily to the M2 phenotype, with or without the addition of interleukin‐4 (IL‐4), compared to a polyethylene glycol diacrylate (PEGDA) hydrogel that lacked the desired cell binding motifs.^[^ ^40^ ^]^

However, the M2 macrophage‐related cytokines, IL‐4 and IL‐13, were reported early on by Anderson et al., to induce the formation of FBGCs by monocyte‐derived macrophages in vitro, although FBGCs have distinct gene expression and cytokine profile which differs from those of the M2 macrophages.^[^ ^30^ ^]^ Lucke et al. found that the CD68^+^ M1 macrophages are negatively associated with the amount of FBGCs after 14 days of implantation, whereas CD163^+^ M2 macrophages were positively related to FBGCs.^[^ ^31^ ^]^ However, Dondossola et al. found that the predominant M1 macrophages in electrospun PCL scaffolds under the dorsal skin of a mouse exacerbated the formation of FBGCs and a fibrotic capsule.^[^ ^37^ ^]^ Witherel et al. recently demonstrated that delivery of IL‐4 and IL‐13 by poly(lactic‐*co*‐glycolic acid) microparticles from GelMA hydrogel implants in mice increased the presence of hybrid M1/M2 macrophages, which resulted in less deposition and less oriented ECM compared to M2 predominant macrophage‐induced ECM deposition. The loosly packed ECM was easier to remodel and cause less fibrotic tissue.^[^ ^36^ ^]^ Mixed and sometimes contradictory results make the role of the macrophage phenotype in the foreign body response inconclusive. However, it is widely accepted that macrophages are a key player in the foreign body reaction and the tissue regeneration process due to their versatility and plasticity. The manipulation of the macrophage phenotype and their role in the tissue regeneration process will be discussed further in Section 4.2.

In the previous literature, the proinflammatory M1 phenotype was considered detrimental to the tissue regeneration process, while the pro‐regenerative M2 phenotype was thought to be beneficial. However, more recent studies have unveiled a delicate balance between the M1 and M2 phenotypes. It is not simply a question of pushing the balance from the M1 to the M2 phenotype in order to achieve the preferred cytokine profile and the desired tissue regeneration outcome. An excessive M1 phenotype leads to chronic inflammation, but its early presence is necessary so as to initiate the downstream inflammatory cascade.^[^ ^41^ ^]^ Disruption of the M1 phenotype during the early stage of biomaterial implantation can delay the inflammatory process and account for cell apoptosis at a later stage.^[^ ^41^ ^]^ On the other hand, the M2 phenotype does promote the healing process. However, immoderate induction of the M2 phenotype leads to fibrotic encapsulation and the fusion of macrophages into FBGCs. In other words, precise temporal control of the macrophage phenotype is important to orchestrate the tissue regeneration process (Figure 3B).

## 2 Vascular Biology

### 2.1 Vascular Anatomy and Physiology

The vasculature is fundamental to the function of all organs, and its dysfunction is related to major diseases such as stroke, myocardial infarction (heart attack), diabetes, and cancer.^[^ ^42^ ^]^ In CAD, as we mentioned earlier in Section 1.1, the stenosis (narrowing) or occlusion (blockage) of the coronary artery reduces the blood supply to the heart muscle, which leads to cardiac malfunction. Human arteries are lined by an inner layer of endothelial cells (ECs) forming the tunica intima, that is in direct contact with the blood and inhibits the initiation of the coagulation cascade. Cell–cell signaling occurs at gap junctions. ECs have inherent adherens junctions with the formation of CD144 (VE‐cadherin), which regulates vascular permeability.^[^ ^18^ ^]^ ECs produce nitric oxide, which regulates vascular hemostasis, dilation, and cell growth.^[^ ^43^ ^]^ Directly underneath the endothelial cell (EC) monolayer, there is a basement membrane or basal lamina, composed mainly of type IV collagen.^[^ ^15^ ^]^

Smooth muscle cells (SMCs) located in the tunica media of the artery wall mediate vaso‐constriction and vasodilation through cellular contraction and relaxation.^[^ ^44^ ^]^ Type I collagen is the major protein component that populates the ECM and provides mechanical support.^[^ ^15^ ^]^ Contractile apparatus proteins produced by SMCs, including *α*‐actin, calponin, smooth muscle myosin (SM‐MHC), and smoothelin, are involved in the contractile function of blood vessels.^[^ ^44^ ^]^ SMCs also play a pivotal role in balancing between ECM secretion and degradation.^[^ ^44^ ^]^ However, under pathological conditions, SMCs can dedifferentiate into a synthetic phenotype, which causes the contractile‐related proteins to be downregulated, and instead they secrete extracellular vesicles, proliferate, and migrate to repair the injury.

The outer layer of the human artery, the tunica adventitia, is a collection of heterogeneous cells including fibroblasts and resident leukocytes such as macrophages, dendritic cells, T cells, B cells, microvascular ECs, pericytes, stem cells, progenitor cells, and nerves.^[^ ^3^, ^34^ ^]^

EC–SMC interactions play an important role in vascular physiology. The ECs provide direct contact and paracrine signaling to the SMCs to regulate their phenotype.^[^ ^7^, ^45^ ^]^ Actively proliferating ECs have been found to stimulate SMC proliferation, whereas confluent ECs do not.^[^ ^44^ ^]^ The vasoactive molecules differentiation, and ECM production.^[^ ^44^ ^]^ The presence of ECs has been shown to elevate the production of elastin from SMCs,^[^ ^44^ ^]^ this being one of the ECM proteins that provide elasticity to blood vessels, which is of vital importance to vascular function.^[^ ^44^ ^]^ Nitric oxide and prostacyclin produced by ECs inhibit SMC proliferation in vitro, while endothelin and platelet‐derived growth factor (PDGF) encourage SMC growth.^[^ ^45^ ^]^ ECs also upregulate the expression of disulfide‐linked homodimer PDGF‐BB and latent TGF‐*β* in cocultured SMCs in vitro, but not the reverse.^[^ ^44^, ^45^ ^]^ The latent TGF‐*β* can only be activated when ECs and SMCs are in close contact.^[^ ^45^ ^]^

In addition to cell–cell interactions, any cell–ECM interaction will also influence cell fate. The degradation of the biomaterial substrate may support cell proliferation. A dense matrix with a limited average pore size can hinder cell division.^[^ ^44^ ^]^ In fact, the pore size distribution and local substrate stiffness are also known to regulate cell behavior.

### 2.2 Vascular Pathology and TEVG Regeneration

#### 2.2.1 Progression of an Atherosclerotic Lesion

Atherosclerosis is a complicated chronic inflammatory disorder that causes blood vessel stenosis (narrowing) and occlusion (blockage).^[^ ^3^, ^46^ ^]^ The progression of vascular lesions and atherosclerosis has been thoroughly reviewed.^[^ ^3^ ^]^ Here, we provide a brief summary of the process.

**Figure 4:**
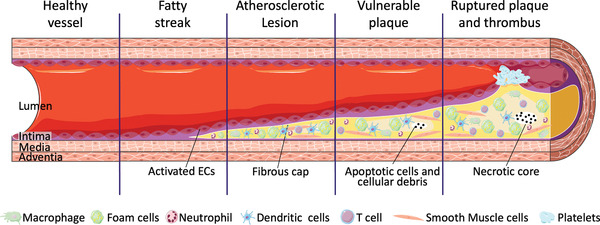
Progression of an atherosclerotic lesion. Reproduced under the Creative Commons Attribution 3.0 Unported License. Copyright 2022, Servier Medical Art.

Atherosclerosis is initiated by the accumulation of lipoproteins and the injury activates ECs as shown in **Figure** **4**. Activated ECs expose intercellular adhesion molecules, such as integrins, selectins, and secreted proinflammatory cytokines, recruit circulating platelets and leukocytes, including monocytes, neutrophils, dendritic cells, and mast cells, to the injury site and subendothelial intimal layer.^[^ ^47^, ^48^ ^]^ As the size of the lesion progresses, lipids continue to build up, and infiltrated monocytes differentiate into macrophages, scavenge lipids, and create foam cells which are fat‐laden M2 macrophages containing low density lipoproteins (LDL).^[^ ^3^ ^]^

At the same time, vascular SMCs and other resident cells begin to infiltrate the intima. These cells continue to produce inflammatory cytokines to recruit even more cells from the circulation and adjacent tissue. This cell aggregation loses its normal function and phagocytosis fails to remove the apoptotic cells within the plaque, resulting in a necrotic tissue core.^[^ ^47^ ^]^

The plaque growth causes thickening of the blood vessel wall and narrowing of the vessel. Once the stenosis reaches 70%, it is considered to be hemodynamically and clinically relevant.^[^ ^2^, ^3^ ^]^ As the lesion continues to progress, a fibrous capsule composed of SMCs and collagen is formed over the plaque. This fibrous capsule contains MMPs whose role is to break down proteins, such as collagen. As the capsule thins due to the enzymatic degradation, the vulnerable plaque lesion may rupture and cause thrombosis in the vessel. The vessel may also try to compensate for the stenosis by dilation, which then increases the risk of vessel rupture.^[^ ^2^, ^3^, ^47^ In most cases, rupture and/or complete occlusion of the vessel will result in acute coronary artery disease and stroke unless there is collateral flow protecting the myocardium from the infarction.^[^ ^2^ ^]^

#### 2.2.2 TEVG Regeneration Process

Understanding the role of vascular cells in the regeneration of vascular tissue is important so as to provide appropriate direction for the design of a TEVG scaffold.

Endothelization is of essential importance to the long‐term patency and function of a TEVG. ECs form a continuous intact monolayer of cells that line the luminal surface of healthy blood vessels. The existence of this healthy endothelium prevents both thrombosis and inflammation and regulates vascular permeability.^[^ ^49^ ^]^ A decellularized natural graft has been reported to activate platelet aggregation immediately after implantation, which subsequently leads to graft occlusion. Endothelial cells produce nitric oxide synthases that have been observed to prevent intimal hyperplasia, the condition when abnormal and excessive cell proliferation and ECM deposition occurs to the vascular inner layer.^[^ ^15^ ^]^ ECs also regulate vasodilation by releasing vasodilators, such as nitric oxide and prostacyclin.^[^ ^49^ ^]^ The lack of a continuous intact endothelium hinders cell–cell signaling at the gap junctions between ECs, particularly at connexins 37, 40, and 43.^[^ ^7^ ^]^ This interruption also induces the activation and migration of vascular SMCs,^[^ ^44^ ^]^ which contributes to intimal hyperplasia and can subsequently progress into atherosclerotic lesions that are associated with vascular narrowing and hardening.^[^ ^7^ ^]^ Activated ECs secrete chemokines promote circulating monocytes directional migration, adhesion, and EC–monocyte interaction, which has been found to be crucial in the early development of atherosclerosis. EC‐derived cytokines, such as angiopoietin 2, C‐X‐C motif chemokine ligand 12 (CXCL12), and colony‐stimulating factor 1, have been found to promote the proliferation and differentiation of perivascular macrophages. In addition, the extracellular vesicles from ECs were also implicated to transfer signaling to perivascular macrophages.^[^ ^34^ ^]^

During the reendothelialization of a vascular graft, the endothelial cells migrate into the implant primarily from the adjacent native vessels.^[^ ^25^ ^]^ EC migration is led by protrusion of filopodia and lamellipodia in the front, followed by the release of the lagging edge of the cell.^[^ ^1^, ^49^ ^]^ The morphology of vascular grafts has long been known to affect the migration of ECs. Parallel aligned nanofibers were implanted to promote EC migration in the longitudinal direction and along the axis of the fibers compared to randomly oriented nonwoven fibrous structures.^[^ ^49^ ^]^ Flow and shear stress also affect EC morphology and function. When ECs are observed with an elongated morphology in the direction of flow, the scaffold resists atherogenesis. Whereas ECs with a cobblestone appearance are usually found under turbulent flow conditions and are associated with atherogenesis.^[^ ^49^ ^]^

The timeframe for achieving full endothelization of a vascular graft varies according to the graft material, the prescribed therapeutics, and the host tissue. An ECM‐based acellular graft in the human arm as a hemodialysis access shunt has been observed to be well revascularized in 16 weeks after implantation.^[^ ^49^ ^]^ In the inferior vena cava (IVC) of a severe combined immunodeficient/beige (SCID/bg) mouse, it was found that a polyglycolic acid (PGA) and poly‐L‐lactide and ‐ε‐caprolactone (PLCL) composite vascular graft pre‐seeded with human bone marrow mononuclear cells (hBMCs) was lined with ECs by 10 weeks and resembled a native vessel after 24 weeks.^[^ ^1^ ^]^ A heparin/VEGF‐immobilized acellular small intestinal submucosa (SIS) TEVG was able to be fully reendothelized within 1 month in the abdominal aorta of a mouse model and the carotid artery of an ovine model.^[^ ^18^ ^]^

The vascular SMC (VSMC) phenotype is another determinant cell type in vascular remodeling and regeneration. A synthetic phenotype that produces ECM is preferred during the early stage of vascular remodeling, and a contractile phenotype is then required to establish hemostasis and vasoactivity. There are also more recently identified phenotypes of VSMCs that affect graft regeneration, including macrophage‐like, osteoblast‐like, myofibroblast‐like phenotypes, and VSMC‐derived foam cells and senescent VSMCs.^[^ ^48^ ^]^ These VSMCs actively interact with other immune cells and are involved in the progression of atherosclerosis and intimal hyperplasia.^[^ ^48^ ^]^ There are also other supporting cells including pericytes and stem cells in the adventitia that contribute to vascular regeneration.

Stem cells are highly involved in the revascularization process. Resident vascular stem or progenitor cells in the vascular intima, media, and adventia, such as endothelial progenitor cells (EPCs), smooth muscle progenitor cells, and mesenchymal stem cells (MSCs) contribute to the revascularization process.^[^ ^50^ ^]^ Kirkton et al. reported how the revascularization process occurred for an engineered graft in a clinical trial. During regeneration, a large number of microvessels form in the adventitia, and ECs create a continuous monolayer on the luminal surface. Over time, the initial CD34^+^ hematopoietic or progenitor cells started to express platelet endothelial cell adhesion molecule‐1 (CD31 or PECAM‐1), indicating the transition from progenitor cells to mature ECs. These endothelial cells were believed to migrate primarily from the surrounding tissue, while a small portion was recruited from the circulating blood.^[^ ^51^ ^]^ It has also been reported that hematopoietic stem cells or bone marrow stem cells can differentiate into VSMCs expressing only *α*‐SMA rather than myosin heavy chains (SM‐MHCs), which are expressed in mature SMCs.^[^ ^3^ ^]^ In an ECM‐based vascular graft implanted in the human arm, Nestin^+^ progenitor cells were observed in the adventitia at 16 weeks and in the media between 18 and 55 weeks. These progenitor cells differentiated into SMC and ECs, which is likely to have contributed to early recellularization and repair of the graft. Along with the increased implantation time, an increased number of MSC‐like CD90^+^ progenitor cells were also observed in the graft.^[^ ^51^ ^]^

In addition to the vascular tissue cells, macrophages play an important role in atherosclerosis and vascular graft remodeling.^[^ ^14^ ^]^ This will be discussed in detail in Section 4.

#### 2.2.3 Cytokines Involved in Atherosclerosis and TEVG Remodeling

Cytokines play a pivotal role in atherosclerosis progression and vascular graft remodeling. Initially there are proinflammatory and atherogenic IL‐1, IL‐6, IL‐18, and TNF‐*α* markers. They are involved in the coagulation cascade, in leukocyte recruitment, endothelial activation, and ECM synthesis.^[^ ^46^ ^]^ At a later point in time anti‐inflammatory and anti‐atherogenic IL‐10, IL‐19, interleukin‐1 receptor antagonist (IL‐1Ra), and IL‐33^[^ ^46^ ^]^ markers are observed. They will be discussed later in this section.

The presence of IL‐1*β* has been observed to promote coagulation, leukocyte‐to‐EC adhesion, and SMC proliferation.^[^ ^52^ ^]^ IL‐1*β* can be activated by a nucleotide‐like receptor protein 3 (NLRP3) inflammasome, which is defined by its pattern recognition receptor (PRR), which oligomerizes to form a procaspase‐1 activating platform in response to DAMPs or PAMPs. There are several members of PRRs that have been confirmed to form inflammasomes: the nucleotide‐binding oligomerization domain, the leucine‐rich repeat‐containing protein 6099 family members such as NLRP3, as well as pyrin.

IL‐1*β* is promoted by cholesterol crystals, neutrophil extracellular traps, tissue hypoxia, turbulent arterial flow patterns as well as by balloon injury and carotid artery ligation.^[^ ^14^ ^]^ These features are often associated with focal development of atherosclerosis.^[^ ^52^ ^]^ For example, the caspase‐1 within NLRP3 inflammasome can cleave pro‐IL‐1*β* into the active form of IL‐1*β*.^[^ ^46^ ^]^

The release of IL‐1 is considered one of the potential therapy targets for cardiovascular disease.^[^ ^53^ ^]^ It has been demonstrated that IL‐1*β* deficient mice are associated with less severe atherosclerosis, and treatment by an antibody against IL‐1*β* can significantly reduce atherogenesis.^[^ ^46^ ^]^ For example, IL‐1*β* inhibition with canakinumab, a monoclonal antibody, decreases the recurrence of cardiovascular events in a randomized clinical trial named CANTOS (Canakinumab Anti‐Inflammatory Thrombosis Outcomes Study).^[^ ^52^ ^]^ Treatment using canakinumab also significantly reduced the level of IL‐6 and the highly sensitive C‐reactive protein in patients. Blockade of IL‐1 reduced leukocyte adhesion and SMC proliferation and attenuated inflammatory cytokines.^[^ ^53^ ^]^

IL‐6 has been implicated as a main contributor to atherogenesis, atherothrombosis, and rheumatoid arthritis.^[^ ^54^ ^]^ The IL‐6 receptor signaling pathway has been proposed as a possible causal pathway for atherothrombosis, since the administration of IL‐6 to apolipoprotein E (ApoE)‐deficient mice resulted in the development of atherosclerosis and the enlargement their lesions.^[^ ^46^ ^]^ It was also found that IL‐1*β* affected atherosclerosis through mediating the IL‐6 signaling pathway.^[^ ^52^ ^]^

IL‐18 is a proinflammatory cytokine and is extensively involved in cardiovascular disease. Receptors for IL‐1*β* and IL‐18 are detected on monocytes, VSMCs and ECs.^[^ ^14^ ^]^ A high level of IL‐18 has been found associated with carotid intima‐media thickening,^[^ ^14^ ^]^ and the administration of IL‐18 to atherosclerosis‐prone apolipoprotein E‐deficient (ApoE^−/−^) mice has been shown to induce atherosclerosis and enlarge lesions, whereas a deficiency of IL‐18 was observed to attenuate atherosclerosis.^[^ ^52^ ^]^

TNF‐*α* is implicated in atherogenesis by upregulating the expressions of intercellular adhesion molecule‐1, vascular cellular adhesion molecule‐1(VCAM‐1) and monocyte chemoattractant protein‐1 (MCP‐1) in the vascular wall, which are known to promote leukocytes recruitment in the early stages of atherosclerosis.^[^ ^55^ ^]^ TNF‐*α* also induces the expression of scavenger receptor class A and the absorption of oxidized LDL by macrophages, which facilitates foam cell formation.^[^ ^55^ ^]^ The avoidance of TNF‐*α* or the administration of agents that reduce TNF‐*α* activity have been found to reduce atherosclerosis and endothelial cell adhesion.^[^ ^46^, ^52^ ^]^

The markers IL‐4 and IL‐10 have long been considered the cytokines that convert macrophages to a pro‐regenerative M2 phenotype. More specifically, IL‐4 can induce an M2a macrophage phenotype that favors regeneration and contributes to vascular stabilization at the anastomosis. However, it is unclear whether an excessive IL‐4 level is likely to contribute to graft failure. It was reported by Nathan et al. that deficiency in IL‐4, IL‐10, and IL‐13 postcoronary artery bypass grafting (CABG) correlated to graft failure,^[^ ^56^ ^]^ yet Bittar et al. suggested that the IL‐4 genotype and serum levels have no relationship to the outcome of a CABG procedure.^[^ ^57^ ^]^ Limited studies have been conducted to evaluate the specific role of IL‐4 in TEVG remodeling and graft stenosis. IL‐10 was found to increase the survival and migration of human EPCs and to upregulate VEGF expression in a murine myocardial infarction model.^[^ ^58^ ^]^

The cytokine IL‐13, that stimulates M2 polarization, partially contributes to the formation of favorable plaque morphology. The administration of IL‐13 to ApoE^−/−^ mice was observed to increase the collagen content and decreased VCAM‐1‐dependent monocyte recruitment. This altered the morphology of the established plaque in the murine model. In fact, the IL‐13 induced M2 macrophages improved the removal of any oxidized LDL in vitro compared to the sample with IFN*γ*‐activated M1 macrophages. Furthermore, a deficiency of IL‐13 in low density lipoprotein receptor knockout (LDLR^−/−^) mice accelerated the atherosclerotic response.^[^ ^59^ ^]^

In an in vivo study myeloid cell‐derived PDGF‐B contributed to a reduction in neotissue formation and retarded polymer degradation of vascular graft scaffold. PDGF‐BB knockout (KO) in a murine model caused macrophage apoptosis and reduced the macrophage population in the tissue engineered vascular graft. The presence of PDGF has also been reported to promote smooth muscle cell migration and proliferation. However, PDGF‐BB alone is not able to prevent graft stenosis in vivo due to complex molecular patterning of the graft remodeling process.^[^ ^60^ ^]^

## 3 The State‐of‐Art of Tissue Engineered Vascular Grafts

TEVGs have been studied for at least two decades, due to the urgent need for a viable small diameter vascular graft as an alternative option to both harvesting autologous vein grafts and implanting a synthetic graft. Small diameter vessels less than 6 mm are required for coronary artery, peripheral popliteal artery, and carotid artery bypass surgery and hemodialysis vascular access. However, the number of scaffold designs that proceed to clinical trials is limited, which means that the translation of a TEVG for clinical vascular bypass or replacement in peripheral arterial disease and hemodialysis vascular access has yet to be realized (**Table** **1**).

**Table 1:** Summary of TEVG studies that involved clinical trials and large animal models

Graft scaffold materials	Scaffold fabrication technique	Graft inner diameter [mm]	Cell sources	Development status	Major results	Research lab or company	References
PGA/PLCL	Weft‐knitted PGA sealed by PCLC foam	–	Autologous bone marrow‐derived mononuclear cells (BM‐MNCs)	Clinical trial (extracardiac‐modified Fontan conduit connecting the IVC to the pulmonary artery in children with single‐ventricle cardiac anomalies)	Japan trial: No acute graft failures or graft‐related deaths Critical stenosis observed in 1 out of 25 patients 3 years after implantation US trial (halted): 3 out of 4 patients developed critical graft stenosis within the first 6 months Early stenosis was treated with angioplasty No additional graft‐related complications	Breuer Lab	Shin'oka et al., 2005; Hibino et al., 2010; Drews et al., 2020
Fibroblast‐deposited ECM	Cell‐assembly extracellular matrix (CAM)	4.8	Autologous human fibroblast	Clinical trial (arteriovenous access)	Function as hemodialysis access for up to 20 months in 5 patients Primary patency was 78% after 1 month postimplantation and 60% after 6 months post implantation.	L'Heureux N Lab	McAllister et al., 2009
Human SMC‐deposited ECM	Cell deposition and decellularization	6	Decellularized	Phase 2 Clinical trial as arteriovenous access and ongoing Phase 3 trial		Niklason Lab/Humacyte	Kirkto et al., **2019**
Human cadaveric SMC‐deposited ECM	Cell deposition and decellularization	6	Decellularized	In vivo (baboon model, arteriovenous access)	Patent for up to 6 months Patency rate was 88% No dilation and change in thickness for 24 weeks No sign of infection, calcification, aneurysmal dilation, and no substantial intimal hyperplasia	Niklason Lab/Humacyte	Dahl et al., **2011**
Allogeneic canine SMC‐deposited ECM	Cell deposition and decellularization	3–4	Autologous ECs	In vivo (canine carotid artery bypass graft)	Patency rate was 83% No sign of infection, calcification, aneurysmal dilation, and no intimal hyperplasia at anastomoses up to 1 year	Niklason Lab/Humacyte	Dahl et al., 2011
Porcine SIS	Decellularized and shaped into a tube	–	Acellular	In vivo (canine model)	Patent for up to 60 days in the carotid artery. Explant vessels showed increased strength due to remodeling. Burst pressures exceeded human vessels (*5600 mmHg).	Lantz	Sandusky et al., 1995; Roeder et al., 2000

Niklason and colleagues have developed a TEVG by seeding allogenic smooth muscle cells from cadaveric donors on degradable PGA tubular scaffolds and culturing them under cyclic radial strain. SMC‐secreted ECM to form the TEVG, and at the same time, the PGA gradually degraded. The graft was then decellularized and stored at 4 °C before implantation. After implantation it showed good short‐term patency (1 month–1 year) in baboon and canine models.^[^ ^61^ ^]^

Such grafts can be engineered with various diameters for different applications, varying from arteriovenous access shunts to coronary or peripheral artery bypass grafts. In the on‐going Phase 2 and Phase 3 clinical trials, similar acellular grafts were used as hemodialysis conduits in patients with end‐stage renal disease.^[^ ^51^ ^]^ The grafts were retrieved from the patients between 16 weeks and 4 years. It was observed that the grafts were populated by mature and circumferentially aligned *α*‐smooth muscle actin positive (*α*SMA^+^) cells in the graft wall. Also, a microvasculature, initiated by CD34^+^/CD31^+^ cells, had formed in the adventitia and was transformed into CD34^−^/CD31^+^ ECs in the media and along the luminal surface of the graft. Nestin^+^ progenitor cells were observed in the adventitia at 16 weeks and in the media between 18 and 55 weeks. These progenitor cells differentiated into SMCs and ECs, which is speculated to contribute to early recellularization and repair of the graft. Along with the increased implantation time, there were an increased number of MSC‐like CD90^+^ progenitor cells being observed in the graft. This study unveiled the changes in cell population and the regenerative process in an ECM‐based vascular graft between 16 weeks and 4 years, which gave us more insights into graft design and graft–cell interactions.^[^ ^51^ ^]^

L'Heureux and co‐workers developed a TEVG by rolling out cell‐assembled extracellular matrix (CAM) sheets deposited by human fibroblasts. These CAM sheets contained over 50 ECM proteins and glycosaminoglycans, including but not limited to, collagen I, collagen VI, thrombospondin‐1, fibronectin‐1, fibrillin‐1, biglycan, decorin, lumican, and versican.^[^ ^62^ ^]^ The fibrous collagen served as the backbone of the sheets to provide the required mechanical performance.^[^ ^63^ ^]^ The autologous CAM‐TEVGs were first evaluated in clinical trials as arteriovenous access devices, which functioned successfully for up to 20 months in high‐risk populations and maintained high patency rates with no access‐related infections.^[^ ^64^ ^]^ However, the high cost of production (greater than $15,000) and the long waiting time (6–9 months) prevented its application in clinical settings.^[^ ^65^ ^]^ L'Heureux and colleagues also studied the use of the allogeneic rolled‐up CAM‐TEVG sheets as shunts in three hemodialysis patients.^[^ ^66^ ^]^ The grafts were cultured from allogeneic fibroblasts, dehydrated, freeze‐dried, and stored at −80 °C prior to implantation. The grafts showed no signs of dilation, degradation or infection during an 11‐month postimplantation period.^[^ ^66^ ^]^

More recently, L'Heureux and colleagues cut their CAM sheets into narrow strips and twisted them into continuous threads to fabricate TEVGs and other tissue engineering constructs in a speedy manner using a variety of textile technologies.^[^ ^67^ ^]^ For example, a woven TEVG was anastomosed as an interpositional graft to a canine carotid artery for one day to show biocompatibility and hemostasis.^[^ ^67^ ^]^ However, the long‐term efficacy of these woven TEVG constructs using CAM threads will need to be evaluated in a larger animal model.

Both Niklason and L'Heureux's approach relies heavily on cells to deposit ECM and form the TEVG, which has introduced the question of whether TEVG production is cell‐species‐dependent. The strength of the TEVG was significantly influenced by the cell species. L'Heureux and colleagues found that human TEVGs were 30‐fold stronger than bovine TEVGs. In fact, the strength of the CAM produced by L'Heureux's particular approach for human fibroblasts was barely reproducible with animal cells. Torres et al. also investigated the reproducibility of CAM cell sheets using animal cells.^[^ ^63^ ^]^ Both human and ovine fibroblasts were able to generate reproducible robust cell sheets, with the human CAM being threefold stronger than the ovine CAM. On the other hand, canine and porcine fibroblasts had difficulty producing cohesive sheets. Serum was identified as one of the critical factors that affected the ability of ECM deposition. And as a result, the optimized culturing conditions were studied and identified to support animal cell‐assembled ECM sheets.^[^ ^63^ ^]^

In comparison to Niklason and L'Heureux, who adopted the deposition of ECM protein as the primary graft material, Breuer and colleagues studied synthetic biodegradable vascular grafts seeded with autologous bone marrow‐derived mononuclear cells (BM‐MNCs) in small^[^ ^68^ ^]^ and large animal models^[^ ^69^ ^]^ as well as in a clinical trial.^[^ ^17^ ^]^ The graft was composed of a PGA knitted mesh sealed by a 50:50 co‐polymer of l‐lactide and *ε*‐caprolactone, designed to degrade within 6 months.^[^ ^17^ ^]^ In a clinical trial in Japan,^[^ ^70^, ^71^ ^]^ a 12–24 mm diameter graft was implanted as an extracardiac cavopulmonary shunt in pediatric patients with a single ventricle physiology. Starting from postoperative Day 2, anticoagulation therapy with aspirin and sodium warfarin was continued for a period of 3–6 months. All the grafts remained patent for the 3–6 months, and during follow‐up, which ranged from 4.3 to 7.3 years (mean 5.8 years), there was no evidence of aneurysm formation, graft rupture, infection or calcification, and no graft‐related deaths.^[^ ^70^ ^]^ More recently a clinical trial in the United States resulted in a high incidence of severe graft stenosis (3 out of every 4 patients) within the initial 6‐month study,^[^ ^17^ ^]^ even though the outcome of their porcine animal model and simulated studies indicated that early stenosis might spontaneously reverse without a clinical intervention. Nevertheless, because of the negative outcome, the clinical trial was stopped at an early stage.^[^ ^17^ ^]^

**Figure 5:**
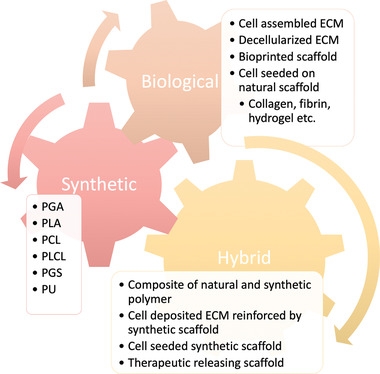
Current material selection and design in constructing a TEVG

**Figure** **5** summarizes the current material selection and therapeutic strategies to fabricate a biodegradable scaffold for TEVG regeneration. Pure protein or polysaccharide‐based biomaterials typically demonstrate excellent cell recruitment and tissue regeneration during immediate (acute) and long term (chronic) inflammatory responses. However, these natural polymeric materials have inferior mechanical properties, limited availability, and variable cost. They lack a reliable and timely supply with uniform characteristics and pose difficulties when scaling‐up production. On the other hand, synthetic materials elicit a strong thrombotic response during the acute stage after implantation. They are associated with a chronic inflammatory response and the development of intimal hyperplasia, which hinder successful healing of the TEVG over the long‐term. Given the increasing evidence of the critical role of the macrophage during the early inflammatory phase, and its ability to regulate the extent of TEVG tissue infiltration and control the regeneration process, the following section in this review will discuss alternative strategies to modulate the macrophage phenotype so as to build a superior and successfully healed TEVG.

## 4 The Role of the Macrophage during the Acute and Chronic Inflammatory Response after TEVG Implantation

In mice, after implantation of a PGA/PLCL TEVG as an IVC interpositional graft, platelet deposition first occurs on the luminal surface and formed a platelet‐rich thrombus within the first day. During the initial 3 days postimplantation, monocytes migrate to the graft and extensively infiltrate the graft wall. This cellular infiltration continues until the graft material completely degrades by 6 months. Early stenosis has been observed during the first 2 weeks after implantation, as the graft wall thickens and the outer diameter remains the same, while the inner diameter decreases. In the event of late stenosis 6 months after implantation, the grafts shrink inwardly, with a decrease of both the inner and outer diameters.^[^ ^25^, ^26^ ^]^

However, with an ePTFE vascular graft, the thrombi that form on the luminal vessel wall are fibrin‐rich, which is different from the platelet‐rich thrombi on the PGA/PLCL vascular graft, indicating that different cellular responses occur to different blood‐contacting surfaces.^[^ ^72^ ^]^

The mural thrombi were infiltrated by macrophages, smooth muscle cells, and fibroblast‐like cells and remodeled into the collagen‐rich neotissue which progressively contributed to the occlusion of the graft. The inflammatory response is resolved quickly in the neotissue composed of ECM proteins, such as collagen and fibrin, but persists against synthetic graft materials such as PGA and PLCL.^[^ ^72^ ^]^ It was also described in an in vitro study that the macrophage polarization to the pro‐regenerative M2 phenotype was mediated by integrin binding.^[^ ^40^ ^]^ These findings supported the use of a natural polymer for TEVG to resolve the issue of chronic inflammation.

**Figure 6:**
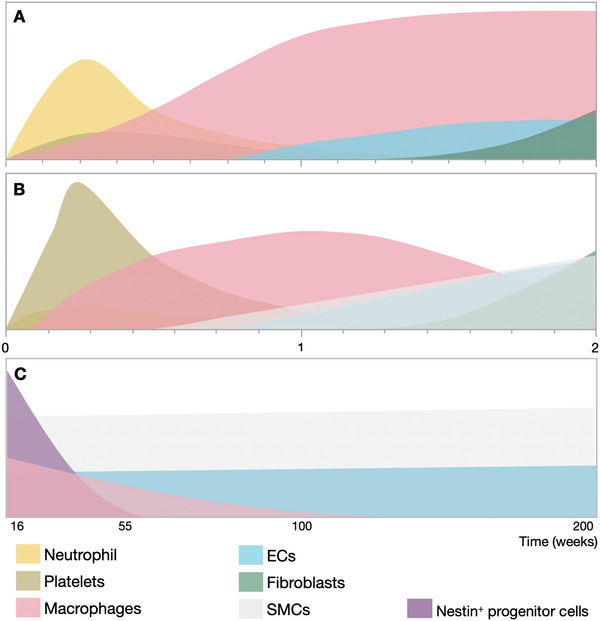
In vivo cell responses following TEVG implantation. A,B) Early response to the (A) PGA/PLCL TEVG was different from that of the (B) luminal neotissue within 2 weeks postimplantation.^[^ ^72^ ^]^ C) Late stage of cell population in an ECM‐based acellular TEVG in human arms 16 weeks to 4 years after implantation. At the late stage, the TEVG was populated by tissue‐forming cells primarily with limited numbers of immune cells. The macrophage population was predominantly CD206^+^ M2 macrophages and contained only a small amount of CD80^+^ M1 macrophages.^[^ ^51^ ^]^ The *x*‐axis represents time in units of weeks. The *y*‐axis represents the relative quantity of cells. EC: endothelial cell. SMC: smooth muscle cell.^[^ ^51^, ^72^ ^]^

Many studies developing TEVGs have placed a priority on promoting endothelialization and regulating the smooth muscle cell phenotype. However, these events happen relatively late after implantation. The early events immediately postimplantation have usually been overlooked. However, these cellular responses create a microenvironment for subsequent tissue regeneration, and therefore are of vital importance to the success of the TEVG. Reinhardt et al. studied the early cellular response to a PLA/PLCL TEVG at the interposition of the abdominal inferior vena cava.^[^ ^72^ ^]^ As shown in **Figure** **6**, a platelet‐rich thrombus formed on the luminal surface shortly after implantation. By Day 1 postimplantation, the graft was primarily infiltrated by neutrophils on the peritoneal side and red blood cells from circulation. During longer periods such as 14 days postimplantation, monocytes, and macrophages increasingly infiltrated from either the peritoneum, the circulation and/or by longitudinal migration. Starting from Day 5, neutrophils are barely detectable and multinucleated giant cells start to be visible. The thrombi were infiltrated by neutrophils, monocytes, SMCs, and fibroblast‐like cells from Day 5 onward. Gradual remodeling with increasing collagen deposition from Day 7, which persistently contributed to graft occlusion.^[^ ^72^ ^]^ Although this timeline of cell infiltration in a murine model is accelerated compared to a more clinically relevant ovine model, the sequence events for cell infiltration remains the same between species. However, the specific mechanism for stenosis remains unclear.^[^ ^72^ ^]^

Given their versatile roles to either sustain or resolve any local inflammatory response, to prevent or promote thrombosis, plaque formation, angiogenesis and tissue regeneration, macrophages have been targeted as an attractive therapeutic agent for cardiovascular disease, as well as a modulator of the biomaterial response and tissue regeneration process.^[^ ^111^ ^]^

### 4.1 Macrophage Infiltration

An increasing amount of evidence has highlighted the critical role of monocytes and infiltrating macrophages in TEVG remodeling and functioning. Monocytes and macrophages are among the first responders after TEVG implantation as we described earlier. Roh et al. reported that after seeding hBMCs on PLCL vascular grafts in an SCID/bg mouse, the hBMCs remained only temporarily in the graft and disappeared within 7 days. Instead, the graft was populated primarily by mouse monocytes, and generated an SMC population within 1 week and endothelium on the luminal layer within 3 weeks.^[^ ^25^ ^]^ It was demonstrated that monocyte recruitment was enhanced by a high level of MCP‐1 released from the hBMCs. MCP‐1 encapsulated in alginate microparticles was embedded in the vascular graft and showed a similar outcome to the hBMC‐seeded vascular graft, which also indicated that MCP‐1‐enhanced monocytes recruitment and promoted TEVG regeneration.^[^ ^1^ ^]^ Although the role of platelets and adaptive immune cells was overlooked by using the SCID/bg animal model, the critical role of monocytes in TEVG regeneration was still demonstrated.

Macrophages actively participate in the angiogenesis process and routine vascular function. For example, they can regulate vascular permeability. The presence of macrophages induces the expression of tight junction related proteins in ECs and the depletion of macrophages disturbs the tight junctions between ECs.^[^ ^34^ ^]^ The conditioned media of primary peritoneal macrophages also promoted the migration of rat aortic vascular SMCs in vitro.^[^ ^73^ ^]^ They are highly involved in angiogenesis by regulating the tip ECs, the stalk ECs, and in the regeneration of a vascular graft by mediating the migration, proliferation and function of ECs, SMCs, and pericytes. This will be discussed more specifically in Section 4.2.

Macrophages are also important at the anastomosis between host vessels and any engineered microvasculature. They physically interact with blood vessels and promote the fusion of the sprouting vessels during angiogenesis.^[^ ^74^ ^]^ Graney et al. observed macrophages wrapping around the vessels and bridging the sprouts. The depletion of host macrophages by administering clodronate in mice significantly reduced macrophage infiltration, preserved more vessel wall, but decreased the level of engineered vessel perfusion.^[^ ^74^ ^]^

Rapid endothelization of a vascular graft also responds to the migration and proliferation of ECs and the differentiation of EPCs from the anastomosis, the surrounding tissue and the circulation, which occurs at a slow pace in large animals and humans.^[^ ^18^ ^]^ M1 macrophages have been shown to accelerate EC and EPC migration, while M2 macrophages reduce migration and yet promote EC proliferation.^[^ ^25^, ^26^, ^74^, ^75^, ^76^, ^77^, ^78^ ^]^ The circulating monocytes can also be captured and participate directly in the endothelization of VEGF‐immobilized vascular grafts.^[^ ^18^ ^]^

Although studies indicate the crucial role of monocytes and macrophages in TEVG remodeling, excessive macrophage recruitment usually leads to neointimal hyperplasia and stenosis of the graft, whereas little macrophage infiltration inhibits neotissue formation and graft remodeling.^[^ ^25^, ^26^, ^75^ ^]^ Thus, modulating a moderate level of macrophage infiltration is of critical importance for the successful integration and function of the TEVG in vivo. Matsuzaki et al. suggested that extensive macrophage influx in the TEVG can lead to graft dilation due to the rapid degradation caused by the macrophages and foreign body giant cells.^[^ ^27^ ^]^ Yet macrophage depletion inhibits graft stenosis. However, macrophage depletion also results in the lack of a functional endothelium, fewer smooth muscle cells and inferior ECM deposition within the graft wall, indicating the critical role of macrophages in successful graft remodeling. Thus, it is an important design requirement to modulate and optimize macrophage infiltration when designing a TEVG.^[^ ^25^, ^26^ ^]^

### 4.2 Manipulating Macrophage Phenotypes

Manipulating the macrophage phenotype has been considered a promising strategy to regulate tissue regeneration. Polarized macrophages are known to release a molecular profile that favors tissue regeneration, which has been more easily achieved compared to releasing a single cytokine or sequential delivery of multiple cytokines to promote certain regenerative outcomes. Although early research focused on polarizing macrophages to either its M1 or M2 phenotype so as to promote either an inflammatory or a regenerative function, recent studies have suggested that an inflammatory response that involves both M1 and M2 macrophages can improve the ultimate tissue regenerative outcome. A balance between these two phenotypes now appears to be more desirable for vascular tissue engineering. Instead of focusing simply on the oversimplified M1/M2 paradigm, it is more important to study the molecules that are involved in preventing graft stenosis and promoting TEVG regeneration.^[^ ^47^ ^]^

#### 4.2.1 M1 Macrophage

In the context of vascular tissue engineering, LPS and IFN‐*γ* induce M1 macrophages to promote EC tip sprouting, which is highly involved in vessel sprouting and early angiogenesis.^[^ ^25^, ^26^, ^74^, ^75^, ^76^ ^]^ ECs cocultured with M1 macrophages upregulated the tip cell phenotype related genes, which involved ADAM metallopeptidase with thrombospondin type 1 motif 9, cathepsin S, C‐X‐C motif chemokine receptor 4, endothelin receptor type B, kinase insert domain receptor, Nidogen 1, neuronal cell adhesion molecule, 6‐phosphofructo‐2‐kinase/fructose‐2,6‐biphosphatase 3, plasminogen activator, urokinase receptor, and Unc‐5 netrin receptor B. It also upregulated genes related to vessel stabilization and lumen formation such as apelin, CD34, and plasminogen activator inhibitor‐1.^[^ ^74^ ^]^ M1 macrophages accelerate EC migration and promote pericyte recruitment in vitro,^[^ ^25^, ^26^, ^74^, ^75^, ^76^ ^]^ which is consistent with their function in supporting tip cells and vessel sprouting.^[^ ^74^ ^]^ They release a series of proangiogenic cytokines and chemokines that facilitate vascular development, such as VEGF, IL‐1*β*, and TNF‐*α*.^[^ ^79^ ^]^ TNF‐*α* was also reported to differentiate EPCs by activating the TNF‐*α* receptor 1 and NF‐*κ*B signaling pathway.^[^ ^78^ ^]^

M1 macrophages also demonstrate other functions such as enhancing the Tie signaling pathway in ECs and promoting EC chemotaxis. They have been shown to improve basement membrane formation and EC–matrix interaction, as indicated by gene ontology enrichment analysis.^[^ ^74^ ^]^

Although the initial participation of M1 macrophages facilitates vascular sprouting, several studies have indicated that the prolonged presence of M1 macrophages adversely affect vascular regeneration. Graney et al. reported that the presence of the M1 macrophage reduced ECs stalk cell phenotype and decreased EC proliferation. The presence of M1 macrophages for a short period of time (1 day) in vitro was able to improve angiogenesis regarding the number and length of vessels and branch points. However, prolonged coculture for 3 days in vitro was found to reduce angiogenesis.^[^ ^74^ ^]^ Lucke et al. also found that CD68^+^ M1 macrophages correlated positively to the presence of Nestin^+^ stem/progenitor cells in the acute inflammation phase (<7 days), while having a negative association with the chronic inflammation phase (>14 days) in PLA meshes in rats.^[^ ^31^ ^]^

M1 macrophage response was also correlated to adverse graft remodeling and stenosis. Inflammatory cell infiltration and chronic inflammation were found to induce arterial thickening and graft stenosis. IL‐1*β*/IL‐18 mediated VSMC‐monocyte crosstalk upregulates VSMC proliferative signals, such as protein kinase B (PKB or AKT), mammalian rapamycin/P70 S6 kinase (mTOR/p70‐S6K), extracellular signal‐regulated protein kinase 1/2 (ERK1/2), and signal transducer and activator of transcription 3 (STAT3). Such activity promotes VSMC proliferation in vitro. Inhibition of IL‐1*β*/IL‐18, by administrating IL‐1Ra‐Fc‐IL‐18bp fusion protein, was shown to reduce VSMC proliferation and wall thickening of vein grafts in a murine model.^[^ ^14^ ^]^ Hibino et al. unveiled that higher levels of proinflammatory cytokines, including C–C motif chemokine ligand 3 (CCL3), inducible nitric oxide synthase (iNOS) and TNF‐*α* in immunocompetent wide type (WT) C.B‐17 mice was related to a greater level of graft stenosis compared to the immunodeficient SCID/bg mice.^[^ ^75^ ^]^ In a clinical study, Herrmann et al re‐endothelized allografts with autologous ECs and implanted them in patients as bypass grafts. The results suggested that the presence of a large population of CD 68^+^ M1 macrophages on the luminal surface of the allograft coincided with the pathological remodeling of the allograft with collagen type I, mainly distributed along the luminal surface, while collagen type IV populated the walls of the vessel.^[^ ^15^ ^]^

#### 4.2.2 M2 Macrophage

M2 macrophages have several subtypes that participate in angiogenesis in different ways, primarily facilitating vascular maturation and anastomosis formation between vessels.

M2 macrophages can promote EC viability, permeability, and proliferation.^[^ ^74^ ^]^ After intratracheally delivered to mice 3 days after cecal ligation and puncture, M2 macrophages were able to promote EC proliferation, recover EC permeability and reduce lung tissue edema. Compared to M0 macrophages, M2 macrophages have been reported to regulate CXCL12, IL‐1Ra, tissue inhibitor matrix metalloproteinase 1 (TIMP1), IL‐4, and CXCL1 in vitro. At the same time, they have been found to regulate granulocyte colony stimulating factor and complement component 5a (C5/C5a) in vivo.^[^ ^80^ ^]^ Both M2a macrophages and their conditioned media were found to improve lung EC viability after an in vitro LPS challenge, indicating that macrophages regulate a paracrine function during tissue regeneration.^[^ ^80^ ^]^

IL4 and IL13 have been reported to induce M2a macrophages to facilitate anastomosis formation by sprouting ECs in vitro. However, the molecular mechanism is unclear.^[^ ^79^ ^]^ M2a macrophages were observed to secrete the highest level of PDGF‐BB, which plays an important role in vascular regeneration. PDGF‐BB can stabilize pericytes as well as promote the migration and differentiation of vascular SMCs and pericytes.^[^ ^74^ ^]^ By removing myeloid cell‐specific PDGF‐B in a murine model, it was found to decrease SMC proliferation and collagen deposition and, at the same time, increase macrophage apoptosis. It was also reported that PGA/PLCL graft degradation and neotissue formation on the luminal surface of the graft slowed down in PDGF knockout mice.^[^ ^60^ ^]^ The M2a macrophages reduced their secretion of matrix metallopeptidase 9 (MMP9) which stimulates angiogenesis. They also released high levels of TIMP3, which can inhibit MMP9 and reduce angiogenesis by preventing VEGF from binding to the VEGF receptor 2.^[^ ^79^ ^]^

It has been shown that IL10 stimulated M2c macrophages regulate vascular remodeling by secreting high levels of MMP9.^[^ ^74^ ^]^ MMP9 is a type of enzyme that degrades elastin, collagen, gelatin (partially hydrolyzed collagen), and other ECM proteins, and thereby creates more room for cell migration, proliferation, and vascular tissue remodeling.

Macrophages also produce reactive oxygen species (ROS) for oxidative degradation of implanted materials.^[^ ^81^ ^]^ Likewise, the degradation of scaffolds can impact in reverse the macrophage response.^[^ ^82^ ^]^ The commonly used synthetic polymers, such as PLA and PGA, produce acidic degradation products. Wu et al. reported that an acidic environment induced by material degradation preferably induced the M2 phenotype, and at the same time reduced macrophage viability.^[^ ^83^ ^]^ M2c macrophages also regulate EC sprouting during angiogenesis, although to a lesser extent than M1 macrophages.^[^ ^74^ ^]^

M2f macrophages are anti‐inflammatory and regulate vessel maturation.^[^ ^74^ ^]^ They can be induced by phagocytosis of apoptotic cells. M2f macrophages are actively involved in SMC and pericyte proliferation and differentiation.^[^ ^74^ ^]^ M2f macrophages secrete transforming growth factor‐beta 1 (TGF‐*β*1), which is involved in endothelial–mesenchymal cell crosstalk to stabilize blood vessels and promote EC migration in vitro and vessel formation in vivo.^[^ ^74^, ^84^ ^]^ Dysregulated TGF‐*β* signaling is highly involved in vascular diseases.^[^ ^85^, ^86^ ^]^ However, inhibition of TGF‐*β* receptor 1 (TGF‐*β*R1) was found to reduce graft stenosis by hindering mesenchymal cell expansion, preventing macrophage activation of the M1 phenotype, and reducing the release of proinflammatory cytokines, such as TNF‐*α*, IL‐12, and IL‐6.^[^ ^87^ ^]^

It is evident that M2 macrophages play a crucial role in neotissue formation and graft remodeling, particularly at the anastomosis between grafts and native vessels. Macrophages with different phenotypes coordinate together and contribute to the angiogenesis and vascular regeneration process. However, it does not benefit the ultimate TEVG outcome by oversimplifying the macrophage response into M1 and M2 phenotypes and then skewing the macrophage polarization to one or the other extreme in the spectrum. Hibino et al. pointed out a high level of Th1‐predominant inflammatory response (M1 response) after graft implantation was related to a greater level of graft stenosis in mice.^[^ ^75^ ^]^ On the other hand, it was demonstrated by Shimizu et al. that a Th2‐predominant inflammatory response (M2 response) induced by a deficiency of IFN‐*γ* receptor in a murine model resulted in severe abdominal aortic aneurysm formation.^[^ ^88^ ^]^

It is also worth mentioning that the timing of the presence of M1 and M2 macrophages is vital to the tissue regeneration process. Spiller and co‐workers showed that early involvement of M1 and M2c macrophages facilitated vascular sprouting in a Matrigel assay in vitro, yet long‐term activation of these phenotypes caused vessel regression.^[^ ^25^, ^26^, ^74^, ^75^, ^76^ ^]^ Initially the presence of M2a macrophages was absent, and they only promoted vascular network formation during later postimplantation periods. For example, Zheng et al. successfully repaired a rat large cranial bone defect by implanting a decellularized bone matrix, and then administering IL‐4 daily injections from Day 3 to Day 7.^[^ ^41^ ^]^ Instead of injecting IL‐4 immediately after implantation, Zheng et al. allowed an early M1 response post implantation, and then promoted an M2 response from Day 3. Although they did not compare the delayed injection of IL‐4 directly with an immediate injection, they still observed that by injecting 10 ng of IL‐4 at the injury site from Day 3 to Day 7 postsurgery, bridging of the bone defect and vascularization of the implanted matrix was successfully achieved in 12 weeks.^[^ ^41^ ^]^ So, while the delivery of cytokines can be controlled by precise daily injections at a bone implantation site, this is more difficult to achieve for a TEVG. Therefore, a biomaterial‐directed delivery of immunomodulatory signals would be a more suitable strategy for TEVG applications.

### 4.3 Source of Macrophage Recruitment

Following an injury, infiltrated macrophages are primarily recruited from perivascular or surrounding tissue as well as from circulating bone marrow derived monocytes. Reinhardt et al. observed in a murine model that the early infiltrated macrophages were primarily located near the outer surface of the graft in the abdominal IVC, suggesting that they most likely migrated from the peritoneal space. The relatively low level of infiltration from the circulation may have indicated the difficulty for cell adhesion directly from the circulation.^[^ ^72^ ^]^

Liu et al. also highlighted the critical role of macrophages recruited from surrounding tissue in the regeneration of vascular tissue.^[^ ^73^ ^]^ They designed a bilayer graft with PCL microfibers (>6 µm) creating a larger average pore size in one layer and nanofibers (<1 µm) generating a smaller average pore size in the second layer. When the microfibers were in the outer layer and the nanofibers were in the inner luminal layer, the graft allowed cell infiltration from the surrounding tissue but limited it from the circulation. On the other hand, when the microfibers were in the inner layer and nanofibers were in the outer layer, the graft accumulated more cells from the circulation than the surrounding tissue. The macrophages were only able to infiltrate the outer layer but not the inner luminal layer, which may suggest the source of these macrophages was primarily from the surrounding tissue. Given that extensive stem cell antigen 1 (Sca‐1) positive stem cells infiltrating the microfiber layer regardless of its location, the source of stem cells is likely to be from both the circulation and the surrounding tissue and was not affected by the average pore or fiber size. After implanting bilayer grafts at the interposition of a rat carotid artery for 1 month, it was found that the graft with inner microfibers and outer nanofibers had a greater patency rate and larger lumen, but a thinner vessel wall and limited numbers of functional ECs and mature SMCs. This result correlated well with the reduced macrophage infiltration from the surrounding tissue.^[^ ^73^ ^]^

Contrary to previous findings, Smith et al. found that the circulating monocytes recruited to their vascular graft contributed to the re‐endothelization of an acellular SIS graft immobilized with heparin and VEGF.^[^ ^18^, ^89^ ^]^ The recruited circulating monocytes first presented only the macrophage markers CD14 and CD 163, but 1 month after implantation, they started to coexpress both macrophage markers and endothelial markers, CD144 and endothelial nitric oxide synthase. These monocyte‐differentiated ECs formed an intact endothelium and presented EC functions, including the formation of a VE‐cadherin positive adherence junction, production of NO, uptake of acetylated LDL, and maintenance of graft patency.^[^ ^18^ ^]^

VEGF‐decorated surfaces are more likely to capture cells expressing VEGF receptor‐1 (VEGFR‐1), which in this case includes primarily circulating monocytes. Monocytes represent >20% of the human peripheral blood mononuclear cells, which is significantly higher than EPCs which represent <0.01%. This situation is likely to contribute to the predominant population of CD14^+^/CD163^+^ M2 macrophages on the graft lumen 1‐week post implantation, instead of ECs or EPCs which express VEGFR‐2. After 1‐month postimplantation, the cells lining the graft lumen express both macrophage markers and endothelial markers and exhibit EC functions.^[^ ^18^ ^]^

Monocytes differentiated toward this EC‐related phenotype through WNT pathway activation via the glycogen synthase kinase 3 (GSK3) antagonist, CHIR‐99021 (CHIR). WNT signaling pathway is known to be activated under shear conditions. In this study, the application of shear stress also enhanced the expression of EC genes, highlighting the critical role of the WNT pathway in monocyte differentiation toward ECs. High shear stress also upregulated arterial markers, Hairy/enhancer‐of‐split related with YRPW motif protein 2 (HEY2) and ephrin type‐B receptor 2 (EphB2), while downregulated the venous markers HEY1 and EphB4.^[^ ^18^ ^]^

These inconsistent monocyte results suggest that recruitment may be due to the different graft compositions. The PCL graft by Liu et al. and PLCL graft by Reinhardt et al. lacked cell binding motifs, and therefore they may not be able to recruit circulating monocytes as efficiently as the VEGF‐decorated graft by Smith et al. The latter graft decorated by VEGF was able to capture circulating monocytes that expressed VEGFR‐1 through a ligand–receptor binding mechanism.

In addition to macrophages, other innate immune cells also contribute to TEVG regeneration and remodeling. The early adhesion of platelets to the damaged endothelium was found to lead to intimal hyperplasia.^[^ ^75^ ^]^ Platelets adhere to the graft luminal surface and rapidly form platelet‐rich mural thrombi after graft implantation. The thrombi are infiltrated by macrophages, SMCs and fibroblast‐like cells and remodeled into neotissue that causes stenosis of the graft.^[^ ^72^ ^]^ Neutral killer (NK) cells were found to encourage arteriogenesis in a murine limb ischemia model. A deficiency in NK cells in the murine model decreased the level of intimal hyperplasia and remodeling of the vascular graft.^[^ ^75^ ^]^

## 5 Immunomodulation in Building Tissue Engineered Vascular Grafts

The traditional immunomodulation approach to tissue engineering tends to avoid activation of the inflammatory response and primarily uses anti‐inflammatory strategies. However, studies have demonstrated that suppressing the inflammatory response retards the tissue regeneration process and hinders the ultimate function of the vascular graft.

Innate immune cells, such as macrophages and neutrophils, are the first responders to the TEVG after implantation. A large number of macrophages infiltrate the TEVG scaffold, and it is challenged to temporally deliver multiple cytokines that regulate the local cell response. However, polarizing the macrophage phenotype can be achieved relatively easily, and the polarized macrophages will release a cytokine profile that favors tissue regeneration by setting up a microenvironment for tissue regeneration in and around the TEVG.

At the present time, the exact immunomodulation conditions have not yet been established for engineering a TEVG. In the following section we review the literature that explores the immunomodulatory function in tissue engineering studies so as to understand the state‐of‐the‐art for the use of biomaterial‐based methods in immunoengineering and tissue engineering.

### 5.1 Biomaterial‐Induced Immunomodulation

#### 5.1.1 Different Physical and Mechanical Properties Drive Different Macrophage Responses

**Figure 7:**
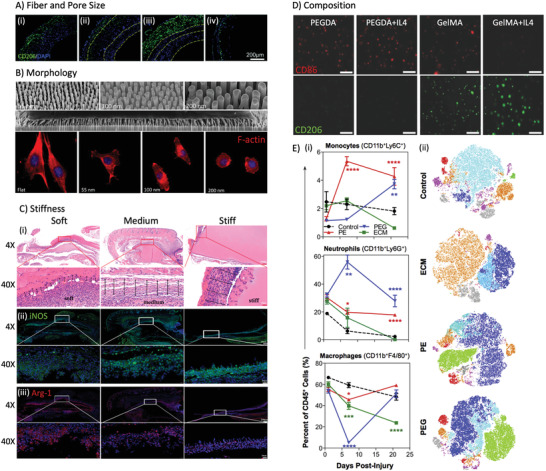
Biomaterial‐induced immunomodulation. A) Thicker fibers on the outer layer of a vascular graft enhance M2 macrophage infiltration (green) in vivo: CD206^+^ macrophages in i) microfiber vascular graft, ii) bilayer graft with outer microfibers and inner nanofibers, iii) bilayer graft with inner microfibers and outer nanofibers, and iv) nanofiber graft. Reproduced with permission.^[^ ^73^ ^]^ Copyright 2022, Royal Society of Chemistry. B) Nanorod array pattern affects macrophage morphology. Reproduced with permission.^[^ ^92^ ^]^ Copyright 2014, American Chemical Society. C) Substrate stiffness impacts macrophage infiltration and polarization: i) histology staining, ii) iNOS staining, and iii) Arg‐1 staining. Reproduced with permission.^[^ ^93^ ^]^ Copyright 2020, American Chemical Society. D) PEGDA hydrogel elicited proinflammatory macrophages (CD86^+^ in red), while the presence of integrin binding sites in GelMA hydrogel enhances pro‐regenerative macrophage polarization (CD206^+^ in green). Reproduced with permission.^[^ ^40^ ^]^ Copyright 2022, Wiley‐VCH GmbH. E) The immune microenvironment is distinct around biologic and synthetic scaffolds in a murine volumetric muscle loss model. i) Myeloid cell populations in control (saline), extracellular matrix (ECM), polyethylene (PE), and poly(ethylene glycol) (PEG). ii) Visualization by flow cytometry of cell population in and around the scaffold at 3 weeks postinjury. Color codes: red: monocytes (CD11b^+^Ly6C^+^), turquoise: macrophages (CD11b^+^F4/80^+^), green: neutrophils (CD11b^+^Ly6G^+^), magenta: dendritic cells (CD11c^+^CD16^−^), blueberry: SAMs (CD11b^+^F4/80^+^CD11c^+^), orange: other myeloid (CD11b^+^), gray: nonmyeloid (CD45^+^CD11b^−^CD11c^−^). Reproduced with permission.^[^ ^94^ ^]^ Copyright 2022, Elsevier.

Fiber size and pore size are two of the more easily controllable physical factors that affect macrophage response. Wang et al. reported electrospun PCL vascular grafts with thick fibers and a large average pore size allowed a moderate level of macrophage infiltration and elicited a stronger M2 macrophage response. Whereas a substrate with thin fibers and a small average pore size restricted macrophage infiltration and induced a predominantly M1 macrophage response. The grafts with thick fibers and a large average pore size also showed superior patency in a rat abdominal aortic model for up to 100 days. It was also reported that the M1 macrophage was observed throughout the whole study, while M2 macrophages arrived as early as Day 7 after implantation and remained for 100 days.^[^ ^90^ ^]^ Consistent with this observation, Liu et al. reported that nanofiber vascular grafts induced more severe inflammation and calcification compared to microfiber grafts.^[^ ^73^ ^]^ The macrophage infiltration and polarization to pro‐regenerative phenotype were enhanced by graft with thicker fiber and larger pore size on the circumferential outer side of the graft (**Figure** **7**A).^[^ ^73^ ^]^ Zhu et al. fabricated prototype vascular grafts by wet spinning PLCL microfibers with circumferential alignment on the inner luminal side and random alignment on the outer surface.^[^ ^91^ ^]^ The circumferential fiber alignment on the luminal layer enhanced cell infiltration and ECM deposition in the circumferential direction. It also contributed to the development of the contractile SMC phenotype, characterized by the staining of SM‐MHCs and accompanied by vascular function in response to vasodilators and vasoconstrictors.^[^ ^91^ ^]^

The morphology and structure of the biomaterial substrate influences the cellular response. However, the specific effect depends on the cell type^[^ ^92^ ^]^ and the substrate's composition and structure.^[^ ^40^ ^]^ Macrophages are able to sense the substrate topography but are not as sensitive as vascular tissue cells. Padmanabhan et al. studied the effect of nano‐topography on the cytoskeleton of macrophages, ECs, and fibroblasts using a bulk metallic glass nanorod array.^[^ ^92^ ^]^ An increase in nanorod diameter, within the range of 55–200 nm, reduced cell spreading, but the degree of the effect was observed to be cell‐dependent. Fibroblasts are most sensitive to pattern changes. They can respond to the pattern change caused by nanorods as small as 55 nm in diameter. For example, with an increase in nanorod diameter, the morphology of fibroblasts appears less extended and elongated. For ECs, when the nanorod array contains rod diameters greater than 55 nm, the extent of EC spreading is significantly reduced^[^ ^92^ ^]^ (Figure 7B).

When comparing a flat substrate surface with 55, 100, and 200 nm nanorod arrays, the larger surface pattern leads to a smaller size of fibroblasts, a higher focal adhesion density, as visualized by paxillin staining, a lower active Rho‐A GTPase level that regulates actin remodeling, and a reduced level of collagen‐I production. This is a two‐edged sword. Limited fibroblast activity, caused by increased topographical features, has limited the extent of fibrosis in the foreign body response, but at the same time, a significant reduction in fibroblast activity can also hinder the process of tissue regeneration. The aspect ratio of nanorods at the surface has also been shown to affect the degree of cell spreading, namely, a higher aspect ratio reduces the extent of cell spreading compared to a lower aspect ratio.^[^ ^92^ ^]^

As stated previously, the response of macrophages to pattern changes in the biomaterial surface is less sensitive than the response by fibroblasts and ECs. Nanorods with a diameter smaller than 200 nm were not able to trigger changes in the macrophage cytoskeleton compared to a flat, unpatterned surface.^[^ ^92^ ^]^ So, while macrophage topography was studied, any changes in the macrophage phenotype caused by the pattern change was not explored in this study.

Surface morphology also influences any macrophage‐mediated material degradation. Wissing et al. studied the effect of fiber size and alignment on scaffold degradation by macrophages. Scaffolds with thicker fibers (6 µm vs 2 µm in diameter) and aligned fibers experienced the most significant macrophage‐driven degradation profile characterized by fiber erosion, chain cleavage, and the presence of malondialdehyde. On Day 4, an aligned thinner fiber scaffold significantly upregulated IL‐6 gene expression in macrophages compared to a random thicker fiber scaffold.^[^ ^81^ ^]^ It was determined that M1 macrophages elicit a significantly more rapid enzymatic degradation profile compared to M2a and M2c macrophages in vitro. However, there was no significant change in phenotype detected among those macrophages cultured on surfaces with different fiber sizes and orientation.^[^ ^81^ ^]^

The mechanical properties of the substrate, such as stiffness or elastic modulus, were also implicated to impact macrophage responses. Zhuang et al. explained that the addition of a stiff GelMA hydrogel at high concentrations elicits a more severe M1 macrophage response, as illustrated by the more intense M1 surface marker staining and the larger quantity of proinflammatory cytokine secretions, which in turn is associated with higher stiffness and smaller average pore size of the hydrogel (Figure 7C). On the other hand, the GelMA hydrogel at lower concentrations results in a larger average pore size and less stiffness, which contributes to more macrophage infiltration and thinner fibrotic encapsulation when implanted subcutaneously in mice.^[^ ^93^ ^]^ Using collagen‐I‐coated polyacrylamide gel substrate, Sridharan et al. also demonstrated that that a soft and medium substrate enhanced anti‐inflammatory gene expression of macrophages in vitro while a stiff substrate enhanced proinflammatory gene expression. The migration and phagocytosis activity of macrophages were also reduced on the stiff substrate compared to the soft and medium substrate.^[^ ^95^ ^]^ It is noteworthy that, in both studies reviewed here, the differences in the macrophage response were ascribed to the bulk stiffness of the hydrogel, which was adjusted by altering the concentration of the gel‐forming polymer. However, it was overlooked and not mentioned that the average pore size of the hydrogel can also be changed by varying the gel concentration. Trappmann et al. pointed out that the cell response was influenced by the local stiffness of the cell‐deposited ECM around the cell. A larger average pore size caused a longer distance between the ECM anchor points and therefore resulted in a more flexible ECM substrate. Whereas a smaller average pore size led to a shorter distance between anchor points and resulted in a stiffer ECM substrate. Cells appeared to respond to the stiffness of the ECM rather than the stiffness of the biomaterial itself.^[^ ^96^ ^]^ Therefore, it might be important to control the pore size in order to implicate the impacts of substrate bulk stiffness on the macrophage responses in the future studies.

Radial compliance, a critical mechanical property for vascular grafts has been found to modulate the macrophage phenotype. A compliant gelatin based vascular graft was reported, by Furdella et al., to have a lower degree of macrophage activation compared to a less compliant PCL graft.^[^ ^97^ ^]^ The compliant vascular graft was also found to favor collagen deposition and a mature SMC pheonotype in the middle layer of the vascular graft.^[^ ^97^ ^]^ However, in this study, the compliant graft was made predominantly from gelatin, while the less compliant graft was made from PCL, making it hard to deconvolute the impact of the material composition from the compliance.

In addition to the surface hardness or elasticity and the bulk compliance of biomaterials, the external mechanical force also influences the phenotype of macrophages. Bonito et al. loaded cyclic strain on the chain‐extended ureidopyrimidinone (UPy)‐modified polycaprolactone (CE‐UPy‐PCL) scaffolds, which were seeded with human peripheral blood mononuclear cells in vitro. It was found that the cyclic strain applied to the material had a tendency to guide the macrophages to a proinflammatory phenotype compared to the static culture.^[^ ^98^ ^]^ Wissing et al. also found that a combination of cyclic stretch and shear stress applied on a macrophage‐seeded scaffold induced a higher level of macrophage activation, evidenced by the elevated expression of both M1 and M2 genes and proteins.^[^ ^99^ ^]^

#### 5.1.2 Chemical Composition Impacts Macrophage Responses

The chemical composition of a scaffold affects the macrophage differentiation and foreign body response. Sadtler et al. showed that different chemical composition of biomaterials elicited distinct immune cell microenvironment in and around them.^[^ ^94^ ^]^ All the biomaterials, including PE, PEG, and ECM, recruited large number of neutrophil and scaffold associated macrophages which was not appear in large quantity in saline treated rat. PE and PEG attracted a high number of neutrophils 3 weeks postimplantation, while ECM recruited a lot of unidentified CD11b^+^ myeloid cells (Figure 7E).^[^ ^94^ ^]^ Reinhardt et al. also reported that, after implantation, ePTFE vascular grafts induce the formation of a fibrin‐rich thrombus, whereas polyester vascular grafts result in a platelet‐rich thrombus in the lumen.^[^ ^72^ ^]^ By comparing the phenotypes of THP‐1 cells encapsulated in PEGDA and GelMA hydrogel in vitro, Cha et al. established that integrin binding sites in GelMA hydrogel facilitated the macrophages polarization to M2 phenotype^[^ ^40^ ^]^ (Figure 7D). PLCL grafts were found to elicit a higher M2/M1 macrophage ratio compared to PCL grafts in vitro.^[^ ^91^ ^]^ Compared to PCL, the superior elasticity, faster degradation, and different composition of PLCL, all seem to correlate with improved remodeling; however, a causative relationship was not fully established. Further investigation on the specific causes that direct positive remodeling would be beneficial to guide future logical and rational TEVG design.

The material degradation rate also influences the rate of macrophage infiltration in vivo. Sugiura et al. found that rapid degradation of the graft's outer layer enhanced cell infiltration and reduced calcification of the graft.^[^ ^100^ ^]^ However, in this study, the rapidly degrading graft was made from a copolymer of lactic acid and glycolic acid, while the slow degrading graft was made from poly(lactic acid). Therefore, it was not clear whether it is the graft degradation profile or the material's chemical composition that influences macrophage infiltration and calcification of the graft.

Ensuring that there is a balance within the graft material design between the pore size, the mechanical properties and the materials degradation profile is challenging yet crucial to the ultimate success of the TEVG. Matsuzaki et al. reported on designing a PLCL/PCL vascular graft, which was implanted in a sheep model.^[^ ^27^ ^]^ The graft was fully endothelized by 8 weeks and remained patent and resistant to dilation and calcification for up to 1 year. However, it was also observed that when the average pore size in the electrospun PCL membrane was only 4 µm, cell infiltration was limited into the scaffold at 1 year. By increasing the average pore size to 15 µm, cell infiltration was significantly improved, yet at the same time the graft was associated with increased hemorrhage during surgery and excessive dilation. The authors did not analyze or explain the reason for the graft dilation with the larger pore size. It was speculated that the excessive dilation may have been due to superfluous macrophage infiltration that reduced the graft integrity.^[^ ^27^ ^]^ It is also possible that the mechanical performance of the graft decreased because the increase in average pore size led to poor durability and subsequent graft dilation. Consequently, continuing studies to optimize material selection and structural design are needed in order to balance cell infiltration with the required mechanical properties.

### 5.2 Biomaterials Carrying Cells with Autocrine, Paracrine or Juxtacrine Signaling

Seeding cells on a scaffold has long been considered beneficial to tissue regeneration. Cells release multiple cytokines and factors after implantation to promote tissue regeneration through a paracrine signaling mechanism. Cell contact has also been reported to be instructive to immune cell response. The seeded cells typically migrate or disappear soon after implantation,^[^ ^1^ ^]^ which indirectly demonstrates the importance of the early events postimplantation in order to ensure a positive tissue regeneration outcome.

Breuer and colleagues investigated seeding BM‐MNCs on hybrid PGA/PLCL TEVGs. Compared to the unseeded control, the BM‐MNC seeded grafts showed elevated patency rates, concentric layers of aligned SMCs, a confluent EC monolayer, and collagen‐rich ECM.^[^ ^68^, ^69^ ^]^ These observations correlate with less platelet adhesion,^[^ ^101^ ^]^ less platelet activation (as measured in terms of platelet‐derived ATP), less macrophage infiltration, and a higher ratio of M2 macrophages in the seeded graft.^[^ ^102^ ^]^ The BM‐MNCs were found to secrete MCP‐1, which enhanced monocyte recruitment during the first week after implantation.^[^ ^1^ ^]^ After 1 week, the seeded BM‐MNCs were no longer detectable in the scaffold. Instead, the host monocytes occupied the graft wall, accompanied by *α*‐SMA^+^ VSMCs. Endothelium lined the luminal surface of the graft by 3 weeks. The infiltrated cells continuously released VEGF throughout the remodeling process.^[^ ^1^ ^]^

It was also found that neither the conditioned media nor the peripheral blood mononuclear cells (PB‐MNCs) were able to substitute for the BM‐MNCs so as to improve graft patency. PB‐MNC seeded grafts implanted in C57BL/6 WT mice did not improve their graft patency compared to grafts incubated in PBS or conditioned media. Compared to PB‐MNCs, BM‐MNCs have been observed to release higher levels of IL‐1*β*, IL‐6, and TNF‐*α*.^[^ ^103^ ^]^ However, the specific role of these cytokines in improving graft patency remains unclear.

MSCs have also been considered a promising candidate for promoting tissue regeneration since they are involved in immunomodulatory interactions. MSCs limit macrophage‐driven fibroblast recruitment, which is believed to reduce excessive fibrosis.^[^ ^19^ ^]^ MSCs have also been implicated in regulating the immune response and promoting tissue regeneration through the release of a wide spectrum of cytokines and chemokines using extracellular vesicles such as small extracellular vesicles (sEVs) and microvesicles. MSC‐derived sEVs have also been implicated to have immunomodulatory and angiogenic responses as well as promote vascular graft patency.^[^ ^104^ ^]^

### 5.3 Immobilization and Encapsulation of Immunomodulatory Cytokines

A number of cell therapy studies have confirmed that the mechanism by which cells primarily influence tissue regeneration is by their paracrine signaling instead of directly contributing to the tissue composition. Therefore, immobilizing or encapsulating cell‐derived signaling molecules on/in the scaffold to generate a sustained release is now considered as an additional approach to promote tissue regeneration and vascularization.

*N*‐hydroxysuccinimide (NHS) is a commonly used cross‐linker to immobilize therapeutic molecules onto a scaffold. Spiller et al. engineered a decellularized bone graft with both physically adsorbed and rapidly releasing IFN‐*γ* and covalently conjugated and slowly releasing IL‐4.^[^ ^108^ ^]^ The IL‐4 and the scaffold were first biotinylated using biotin‐sulfo‐LC‐LC‐NHS (*N*‐hydroxysuccinimide) and conjugated together using streptavidin. This design allowed an immediate release of IFN‐*γ* within 48 h postimplantation and a relatively sustained release of IL‐4 over 6 days. The results indicated that the initial burst release of IFN‐*γ* without the addition of IL‐4 promoted early M1 macrophage polarization. This significantly increased blood vessel density in the bone graft compared to the other groups. Yet the sequential release of IFN‐*γ* and IL‐4 did not significantly improve vascularization of the scaffold. Even though the release of IL‐4 persisted for 6 days, around 50% of the IL‐4 was released rapidly during the early acute phase, which may have resulted in a mix of M1 and M2 macrophages immediately postimplantation. In addition, it is worth noting that the loading efficiency of both IFN‐*γ* and IL‐4 was very low. Therefore, improving the efficiency of protein immobilization and controlling more accurately the release profile are key issues that are critical to the success of this methodology.^[^ ^108^ ^]^

Heparin is a naturally occurring glycosaminoglycan that is typically used as an anticoagulant. It is negatively charged, resulting in its affinity to a wide range of cytokines. Therefore, it has also been used extensively as an immobilizer to connect immunomodulatory cytokines to a TEVG and mediate its immune response. This is especially beneficial for vascular tissue engineering because of its dual effect of both preventing thrombosis and conjugating immunomodulatory cytokines. Matsuzaki et al. blended heparin with PLCL in a methylethylketone/acetone/ethanol solvent to locally deliver heparin from the PLCL sponge/electrospun PCL bilayer vascular graft. Their objective was to protect the graft from thrombosis before a continuous intact endothelium was formed. Although 97% of the heparin was released within the initial 24 h, the incorporation of heparin still significantly reduced platelet adhesion and thrombosis formation when evaluated in a sheep carotid bypass model. It also reduced the thrombotic occlusion rate for the graft from 60% to 0% within one week of implantation.^[^ ^27^ ^]^

**Figure 8:**
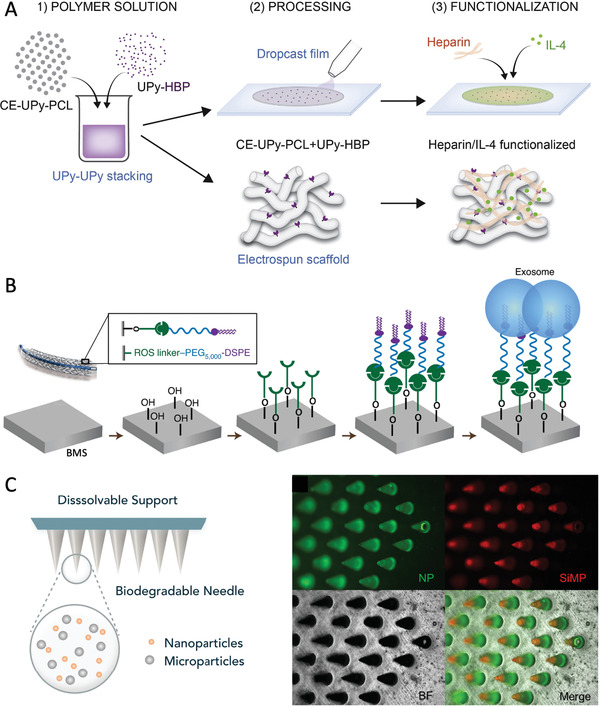
Strategies of delivering immunomodulatory cytokines to modulate macrophage response. A) Heparin and IL‐4 were immobilized on the electrospun scaffolds to modulate macrophage response and promote vascular regeneration. Reproduced with permission.^[^ ^105^ ^]^ Copyright 2022, Elsevier. B) The elution of MSC exosomes on a bare metal stent (BMS) decreased platelet and monocyte adhesion. It also upregulated M2‐related gene expression in the rat aorta, which might contribute to the enhanced reendothelialization and reduced SMC migration. Reproduced with permission.^[^ ^106^ ^]^ Copyright 2022, Springer Nature. C) A microneedle patch containing tetracycline‐loaded nanoparticles and IL‐4/TGF‐*β*‐loaded silica microparticles was able to provide a rapid release of antibiotics and a sustained release of IL‐4 and TGF‐*β*. Reproduced with permission.^[^ ^107^ ^]^ Copyright 2022, Elsevier.

Bonito et al. engineered a heparin‐IL‐4 conjugated PCL scaffold that induced a high ratio of M2/M1 macrophages in vitro. The scaffold was fabricated by drop casting a mixture of UPy‐modified chain extended PCL and UPy‐modified heparin binding peptide (**Figure** **8**A). Heparin and IL‐4 were premixed and incubated with the scaffold to complete the immobilization. The scaffold was able to upregulation the expression of TGF‐*β*1 and MMP‐9, both of which are key players in graft stenosis and remodeling.^[^ ^105^ ^]^

Koobatian et al. developed an acellular SIS graft immobilized with heparin and VEGF that demonstrated high patency, a continuous intact endothelium, extensive cell infiltration, and ECM remodeling in the vascular wall of an ovine animal model. The heparin was immobilized on the SIS graft using 1‐ethyl‐3‐(3‐dimethylaminopropyl)carbodiimide (EDC) and NHS, and the VEGF was then attached to the scaffold via the heparin. Interestingly, the SIS graft modified by heparin alone occluded 2 days postimplantation, indicating that the addition of an anticoagulant by itself is not adequate to improve graft patency. It also underscores the important role of VEGF in maintaining graft patency.

Wei et al. designed the release of MSC‐derived sEVs from an electrospun PCL vascular graft to enhance vascular regeneration. They also cross‐linked heparin onto the PCL graft surface using ethylenediamine, EDC, and NHS. So in effect, heparin was used both as an anticoagulant as well as a linkage molecule for MSC‐derived sEVs. Following implantation of the graft in the abdominal aorta of hyperlipidemic mice for 3 months, the heparin and heparin–sEV loaded grafts showed greater patency compared to bare PCL control. In addition, the sEV samples also promoted tissue regeneration and prohibited calcification. The sEV‐loaded graft developed more neotissue on the luminal surface compared to the untreated pristine PCL and heparin‐functionalized PCL control grafts. Although all grafts were lined by a confluent endothelium, the neotissue developed in the sEV‐loaded graft contained a higher number of mature and contractile SMCs, which was not observed in the control grafts. Surprisingly, the heparin‐functionalized graft showed a significantly higher degree of calcification. However, the addition of sEV was able to reduce the level of calcification caused by the heparin.^[^ ^104^ ^]^

It was also observed that the grafts loaded with sEVs had fewer M1 macrophages and more M2 macrophages. In a separate in vitro cell culture study, grafts loaded with sEVs were able to polarize macrophages to the M2 phenotype at the gene expression level. In fact, the presence of the sEVs was also associated with a reduction in the expression of pro‐osteogenic‐related genes TGF‐*β*1 and Wnt family member 10B in the macrophages. Although there was no evidence that there was a direct causative effect, the authors correlated the more abundant M2 macrophage phenotype to the superior vascular outcome; namely, the improved patency rate, neotissue formation with the regeneration of continuous endothelium and contractile SMCs.^[^ ^104^ ^]^

Hu et al. eluted MSC exosomes on the bare metal stent for vascular intervention to reduce inflammation and promote healing after stent deployment.^[^ ^106^ ^]^ The stent was conjugated with 1,2‐distearoyl‐sn‐glycero‐3‐phosphoethanolamine to immobilize MSC exosomes through a linker that is sensitive to ROS levels (Figure 8B). The exosomes released in response to the elevated level of ROS caused by the mechanical injury after stenting in the blood vessel. The exosome‐eluting stent decreased the platelet and monocyte adhesion in vitro, as well as promoted EC proliferation, and reduced SMC migration. In a rat aorta model, the exosome‐eluting stents elicited less inflammation and formed less neointima and stenosis compared to bare metal stents.^[^ ^106^ ^]^

Methacrylation and thiolation of heparin have also been proposed so as to chemically conjugate heparin to photo‐cross‐linkable biomaterials such as GelMA. Methacrylation of heparin can be achieved by reacting heparin with methacrylic anhydride. Thiolation of heparin can be realized by reacting heparin with EDC, hydroxybenzotriazole, and cysteamine. Both modifications to heparin allow photo‐cross‐linking and attachment of heparin to GelMA hydrogel. Brown et al. compared these two methods and found that thiolated heparin preserved the anticoagulant property more effectively than methacrylated heparin. Yet at the same time, the retention rate was significantly higher for the methacrylated heparin.^[^ ^109^ ^]^

Another interesting mechanism that has demonstrated a positive outcome is to involve biological markers that are responsible for phagocytosis. Gauthier et al. designed a liposome surface modification with 10% phosphatidylserine (PS), which is a phospholipid present on apoptotic cell membranes.^[^ ^110^ ^]^ The presence of the PS mimicked the apoptotic signal and induced efferocytosis, or the phagocytic removal of apoptotic cells, which resulted in an uptake of the liposome by the macrophages. The PS‐modified liposome was utilized to locally deliver the anti‐inflammatory drug dexamethasone preferentially to macrophages, which in turn induced a pro‐regenerative macrophage phenotype. So delivery of these biological markers increased efferocytosis activity and the secretion of thrombospondin 1, decreased the release of proinflammatory cytokine IL‐6 and TNF‐*α*, and increased the release of anti‐inflammatory cytokines TGF‐*β*1 and IL‐10.^[^ ^110^ ^]^

In addition to immobilization of cytokines on the scaffolds, encapsulating cytokines in micro‐ or nanoparticles is also an emerging strategy. Zhang et al. encapsulated IL‐4 and TGF‐*β* in heparin modified silica microparticles and added to GelMA microneedle patch for sustained codelivery of IL‐4 and TGF‐*β* to promote macrophage polarization to the pro‐regenerative phenotype (Figure 8C).^[^ ^107^ ^]^ The delivery of IL‐4 significantly downregulated proinflammatory genes (iNOS and IL‐1*β*) and upregulated pro‐regenerative genes (MRC1 and ARG‐1) in murine bone marrow‐derived macrophages in vitro. The IL‐4/TGF‐*β* codelivery microneedle patch in a rat periodontitis model also downregulated the inflammatory gene TNF‐*α* and upregulated anti‐inflammatory and pro‐regenerative gene IL‐10, RUNX2, and BMP‐2.^[^ ^107^ ^]^

Overall, carrying immunomodulatory cytokines with a TEVG scaffold is a promising method to modulate the macrophage response and promote the regeneration of a TEVG. The quantity of cytokines loaded onto a scaffold needs to be determined with precision. Insignificant quantities might not be helpful, yet superfluous cytokine release can cause negative effects as discussed previously. In addition, because of the degradability and sensitivity of cytokines, in order to make them available off‐the‐shelf, the storage conditions, and sterilization methods of cytokine‐carrying TEVG scaffolds needs to be carefully studied and evaluated.

## 6 Challenges and Opportunities

TEVGs have been extensively studied over the past decades, yet limited success has been achieved. Although a few groups have proceeded to a human clinical study, there is still a long way to go before we can bring a viable TEVG to the market and the clinic to fulfill the need of patients for coronary, peripheral, carotid artery bypass surgery, and arteriovenous access.

The principle of a TEVG is to engineer a biodegradable scaffold and implant it as a bypass graft. The TEVG serves as a temporary scaffold to withstand mechanical loads, to interact with the native environment and boost tissue recruitment and regeneration, especially facilitating fast endothelialization and repopulation of mural cells in the blood vessel wall. Biomaterials, such as collagen, gelatin, elastin, show extraordinary biological performance but lack structural integrity and mechanical robustness to be used as a vascular graft. Synthetic materials, such as extensively studied PGA, PLA, PCL, PLCL, tend to have excellent mechanical properties, but their lack of cell‐binding motifs elicit adverse cellular response such as chronic inflammation and retard the tissue regeneration process. Therefore, optimizing graft material design is one of the remaining challenges in the study of TEVGs.

Given that all biomaterials elicit more or less an immune response, which might involve some platelet activation and inflammation immediately after implantation, it is impossible to circumvent the immune response. Therefore, modulating immune cell response to the biomaterials is one of the approaches to ameliorate tissue regeneration outcome. And it has been demonstrated that monocytes and macrophages are highly involved in the regeneration process of synthetic TEVGs.^[^ ^18^, ^25^, ^26^, ^39^, ^72^, ^75^ ^]^ Among the immune cells' response to biomaterial implants, macrophages have been shown to infiltrate into and respond to the material early in the process and present plasticity and multiple roles in the foreign body response and tissue regeneration processes. Therefore, macrophages have attracted lots of interest in the area of tissue engineering. Extensive studies have been conducted to elucidate different phenotypes of macrophages and their role in the tissue regeneration process. Yet limited attempts have been made to modulate macrophages in building a TEVG.

Researchers have limited knowledge about the very early response to different biomaterials implanted as blood vessels. The specific mechanisms of graft thrombosis and stenosis caused by the composition and construction of biomaterials remain elusive, making it difficult to identify therapeutic targets to optimize the design of the graft material and structure. Continuing to study how material properties influence the immune cell response is of the key importance to enrich our understanding of the interaction between immune cells and biomaterials.

## Conflict of Interest

The authors declare no conflict of interest.
